# Arabidopsis ribosomal RNA processing *meerling* mutants exhibit suspensor-derived polyembryony due to direct reprogramming of the suspensor

**DOI:** 10.1093/plcell/koae087

**Published:** 2024-03-21

**Authors:** Honglei Wang, Luca Santuari, Tristan Wijsman, Guy Wachsman, Hannah Haase, Michael Nodine, Ben Scheres, Renze Heidstra

**Affiliations:** Cluster of Plant Developmental Biology, Laboratory of Cell and Developmental Biology, Wageningen University & Research, Droevendaalsesteeg 1, 6708 PB Wageningen, The Netherlands; Cluster of Plant Developmental Biology, Laboratory of Molecular Biology, Wageningen University & Research, Droevendaalsesteeg 1, 6708 PB Wageningen, The Netherlands; Cluster of Plant Developmental Biology, Laboratory of Cell and Developmental Biology, Wageningen University & Research, Droevendaalsesteeg 1, 6708 PB Wageningen, The Netherlands; Molecular Genetics, Department of Biology, Faculty of Science, Utrecht University, Padualaan 8, 3584 CH Utrecht, The Netherlands; Cluster of Plant Developmental Biology, Laboratory of Molecular Biology, Wageningen University & Research, Droevendaalsesteeg 1, 6708 PB Wageningen, The Netherlands; Cluster of Plant Developmental Biology, Laboratory of Molecular Biology, Wageningen University & Research, Droevendaalsesteeg 1, 6708 PB Wageningen, The Netherlands; Cluster of Plant Developmental Biology, Laboratory of Molecular Biology, Wageningen University & Research, Droevendaalsesteeg 1, 6708 PB Wageningen, The Netherlands; Cluster of Plant Developmental Biology, Laboratory of Cell and Developmental Biology, Wageningen University & Research, Droevendaalsesteeg 1, 6708 PB Wageningen, The Netherlands

## Abstract

Embryo development in Arabidopsis (*Arabidopsis thaliana*) starts off with an asymmetric division of the zygote to generate the precursors of the embryo proper and the supporting extraembryonic suspensor. The suspensor degenerates as the development of the embryo proper proceeds beyond the heart stage. Until the globular stage, the suspensor maintains embryonic potential and can form embryos in the absence of the developing embryo proper. We report a mutant called *meerling-1* (*mrl-1*), which shows a high penetrance of suspensor-derived polyembryony due to delayed development of the embryo proper. Eventually, embryos from both apical and suspensor lineages successfully develop into normal plants and complete their life cycle. We identified the causal mutation as a genomic rearrangement altering the promoter of the Arabidopsis *U3 SMALL NUCLEOLAR RNA-ASSOCIATED PROTEIN 18* (*UTP18*) homolog that encodes a nucleolar-localized WD40-repeat protein involved in processing 18S preribosomal RNA. Accordingly, root-specific knockout of *UTP18* caused growth arrest and accumulation of unprocessed 18S pre-rRNA. We generated the *mrl-2* loss-of-function mutant and observed asynchronous megagametophyte development causing embryo sac abortion. Together, our results indicate that promoter rearrangement decreased UTP18 protein abundance during early stage embryo proper development, triggering suspensor-derived embryogenesis. Our data support the existence of noncell autonomous signaling from the embryo proper to prevent direct reprogramming of the suspensor toward embryonic fate.

## Teaser

Arabidopsis *meerling-1* mutant exhibits suspensor-derived polyembryony due to delayed processing of the 18S preribosomal RNA, resulting in reprogramming of the suspensor toward embryonic fate.

**IN A NUTSHELL**

**Background:** In sexually reproducing land plants, a seed normally contains a single mature embryo originating from a single fertilized egg cell. Embryo development then generates the embryo proper and the supporting extraembryonic suspensor, which later degenerates as embryo development proceeds. In *Arabidopsis thaliana*, this rule of “one seed, one embryo” is executed well. However, the suspensor maintains embryonic potential until the globular stage and can form embryos in the absence of the developing embryo proper, suggesting a signaling pathway inhibiting the embryonic potential of suspensor.

**Question:** What is the genetic basis of zygotic polyembryony and which are the molecular mechanisms involved?

**Findings:** We report a recessive hypomorphic mutant, *meerling-1* (*mrl-1*), with high penetrance suspensor-derived polyembryony due to the delayed development of the embryo proper. We identified the causal mutation as a genomic rearrangement altering the promoter of the Arabidopsis U3 SMALL NUCLEOLAR RNA-ASSOCIATED PROTEIN 18 (UTP18) homolog, thereby decreasing protein abundance during early-stage embryo proper development. UTP18 encodes a nucleolar localized WD40-repeat protein involved in processing 18S pre-ribosomal RNA. Complementation analysis indicated that UTP18 functions non-cell autonomously to prevent suspensor reprogramming toward embryonic fate. Together, our results provide detailed molecular evidence in support of active signaling between developmental progression of the embryo proper and suspensor to sustain the development of a single dominant embryo.

**Next steps:** The question that remains is to the nature of the signaling between the embryo proper and suspensor? Future work will focus on identifying the genes required suppress suspensor reprogramming. The *mrl* mutants, because of the high penetrance of polyembryony, provide an excellent tool for unraveling these signaling components.

## Introduction

In sexually reproducing land plants, a seed normally contains a single mature embryo originating from a single fertilized egg cell, the zygote. Nevertheless, certain plant species can produce two or more embryos from one seed, termed polyembryony (Lakshmanan and Ambegaokar 1984). Polyembryos can emerge from nonzygotic (maternal) tissue without fertilization, a process called apomixis, or by cleavage from sexual-derived zygotic tissue (Lakshmanan and Ambegaokar 1984). Apomixis has attracted much attention since it allows the maintenance of commercially valuable traits that segregate during sexual reproduction of hybrid plants (Marimuthu et al. 2011; Kelliher et al. 2017; Wang et al. 2019; Zhong et al. 2019). Natural polyembryony by apomixis in citrus is a well-known example and was reported to be first described by Van Leeuwenhoek in 1719 (Lakshmanan and Ambegaokar 1984). Natural zygotic polyembryony is common in gymnosperms and occurs during early embryo development. For example, in *Pinus* species the initial proembryo divides to give rise to four embryos. Only one of the embryos develops further into a mature embryo, while the others are degraded by programmed cell death (Filonova et al. 2002; Merino et al. 2016). Other species show zygotic polyembryony derived from the usually quiescent suspensor that connects the embryo to the maternal tissues (Lakshmanan and Ambegaokar 1984). However, little is known about the genetic basis of zygotic polyembryony in plants and the molecular mechanisms are largely unexplored.

Whereas a universal cell division pattern during plant embryo development does not exist, the model plant Arabidopsis (*Arabidopsis thaliana*) presents an almost invariable sequence of early embryonic cell cleavages and therefore lends itself well for studies on embryonic patterning (West and Harada 1993; Jürgens and Mayer 1994). During Arabidopsis zygotic embryogenesis, an asymmetric division of the zygote forms a smaller apical cell and large basal cell. The apical cell is the major founder of the embryo proper and undergoes divisions to form the majority of tissues in the mature embryo and germinated seedling. The basal cell undergoes limited cell division and in large part differentiates into the suspensor. The embryo is connected to maternal tissue by the suspensor for structural support and nutrient exchange (Kawashima and Goldberg 2010; Peng and Sun 2018). The topmost suspensor cell generates the hypophyseal cell that becomes part of the embryonic and seedling root (Jürgens and Mayer 1994; Scheres et al. 1994; ten Hove et al. 2015). The remaining suspensor cells undergo programmed death at the heart stage of embryonic development (Bozhkov et al. 2005).

In Arabidopsis, the rule “one seed–one embryo” is executed well. However, the discovery of the *twin1* (*twn1*) mutant, which displayed polyembryony with a 9% penetrance, showed that this rule can be broken (Vernon and Meinke 1994). Interestingly, the supernumerary embryos developed from the suspensor. This led to the hypothesis, developed in the 1990s, that the suspensor has a potential to form an embryo, but this potential is suppressed by the developing embryo proper (Schwartz et al. 1994; Vernon and Meinke 1994). Since then, this hypothesis has been supported by evidence from several studies. For example, disturbing the development of the embryo proper by genetic ablation can induce cell proliferation in suspensor cells, resulting in at least partial switching to embryonic fate (Weijers et al. 2003). More direct evidence came from laser ablation studies whereby damage of the embryo proper triggered suspensor cells to develop into an embryo (Gooh et al. 2015; Liu et al. 2015). Also, twin embryos were induced by reducing auxin activity through ectopic expression of a stabilized version of INDOLE-3-ACETIC ACID INDUCIBLE 12/BODENLOS (IAA12/BDL) (Rademacher et al. 2012; Radoeva et al. 2020). Additional mutants showing suspensor-derived polyembryony have been reported that may provide further insight into the genetics behind the communication mechanism between embryo proper and suspensor. In *suspensor* (*sus*) and *raspberry* mutants, the suspensor-derived embryo-like structures undergo limited cell division but are inviable (Schwartz et al. 1994; Yadegari et al. 1994). Hypomorphic allele combinations of *iyo*, mutated in a positive regulator of transcriptional elongation that is essential for differentiation onset, can develop ectopic embryos from the suspensor that successfully germinate. However, the resulting plants cannot complete their life cycle to produce seeds (Sanmartín et al. 2011). Finally, the *twn2* mutation, a T-DNA insertion in the 5′ flanking region of a valyl-tRNA synthetase gene, caused early proembryo cell division arrest accompanied by suspensor produced twin embryos (Zhang and Somerville 1997). Shared between all these mutants producing suspensor-derived embryos is the arrest of the embryo proper. The only exception is *twn1*, in which both proembryo and suspensor can develop into normal healthy plants (Vernon and Meinke 1994), but for which the causal gene remains unclear.

Here we identify and characterize a recessive hypomorphic Arabidopsis mutant, *meerling-1* (*mrl-1*), with high penetrance polyembryony. Supernumerary embryos originate from the suspensor and together with the proembryo can grow out to form healthy adult plants. We identified the causal gene, which encodes a conserved WD40-domain U3 small nucleolar RNA-associated protein 18 (UTP18) homolog, and revealed its function in the cleavage of the 18S preribosomal RNA. A *UTP18* knock-out allele (*mrl-2*) showed arrested female gametophyte development which resulted in ovule abortion. Complementation analysis indicated that UTP18 functions noncell autonomously to maintain suspensor identity. Together our results support the hypothesis that the embryo proper inhibits the developmental potential of suspensor cells, not as a response to physical perturbation but as a monitoring system for developmental progression.

## Results

**Figure 1:**
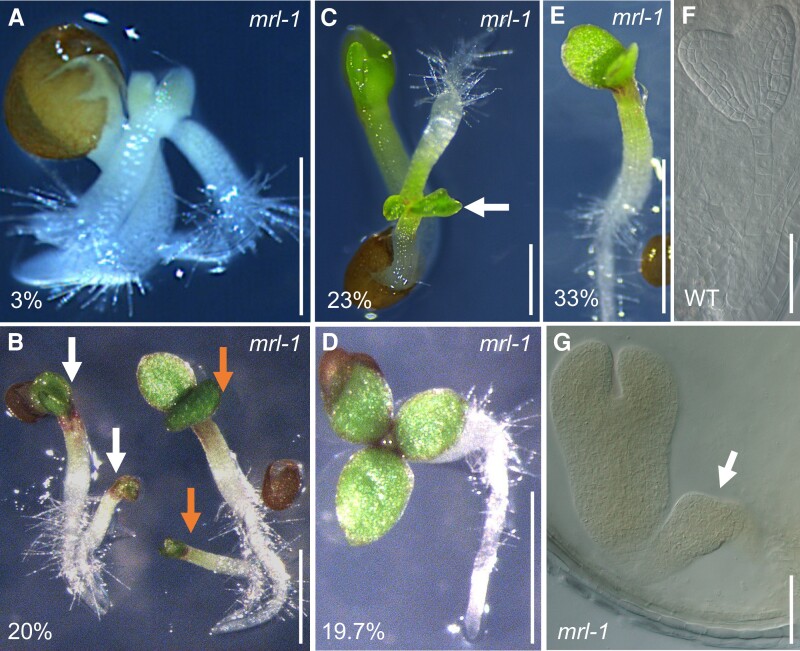
. Polyembryo and polycotyledon phenotypes displayed by the *mrl-1* mutant. **A to C)** Multiple seedlings germinating from a single seed, exemplified by triplets (**A**, **C**) and twins (**B**, arrows). Triplet in (**C**) shows two seedlings fused at the shoot (arrow). **D)** Tricotyledon seedling. **E)** Wild-type-like seedling. Percentages indicate phenotype penetrance. **F)** WT heart stage embryo supported by a single file of suspensor cells. **G)***mrl-1* heart shape embryo with ectopic embryo structure (arrow) developing from the suspensor. Scalebars in (**A**) is 2.5 mm, in (**B** to **E**) is 10 mm, in (**F**, **G**) is 50 *µ*m.

Following *Agrobacterium tumefaciens* (Agrobacterium)-mediated transformation of the *pCB1* construct, carrying norflurazon and phosphinothricin resistance cassettes (Supplementary Fig. S1A) into the transgenic Arabidopsis ecotype Nossen (No-0) carrying *HSP18.2_pro_:CRE* (HCN) (Sieburth et al. 1998; Heidstra et al. 2004), we observed that multiple seedlings germinated from single seeds in the progeny of line T2-10 (Fig. 1A). We next performed a series of crosses and selfings with these polyembryo-derived plants and found that the phenotype was stably inherited as a recessive trait. Since we observed more than two seedlings germinating from a single seed (Fig. 1A), we named this mutant *meerling* (*mrl-1*) (“multiple birth”). However, the phenotype could not be linked to the presence of the *pCB1* T-DNA based on the observation of norflurazon sensitive seedlings displaying polyembryony (Supplementary Fig. S1B) and the absence of T-DNA hybridizing fragments in the DNA of their progeny tested by DNA gel blot analysis (Supplementary Fig. S1, C and D).

### *mrl-1*, a polyembryonic mutant

To characterize the polyembryo phenotype, we germinated seeds of five homozygous T3 lines (Supplementary Data Set 1) and observed a penetrance of 33% to 53% for polyembryonic seeds and 13% to 26% for tricotyledon seedlings. The remainder germinated as single WT-looking seedlings. To determine the stability of this phenotype, we carefully re-examined one of these lines after several generations of inbreeding (Supplementary Data Set 1). We germinated 228 seeds of inbred *mrl-1* line and observed almost half of the seeds (105/228) producing multiple seedlings (Fig. 1, B and C). Of these polyembryonic seeds, less than half (45/105) produced twins and the remainder (60/105) produced triplets. The majority of triplets (53/60) comprised one complete seedling with the other two seedlings fused together in the junction of the cotyledons (Fig. 1C), whereas a few triplets were separated upon germination (7/60). Together, these results showed that the penetrance of the *mrl-1* polyembryony phenotype is around 46%. Apart from the polyembryo phenotype, about 20% of the seedlings showed polycotyledony, with the majority displaying three cotyledons (45/228) (Fig. 1D). Throughout these experiments we observed two seedlings germinating with four cotyledon-like structures and one with double shoots supported by a single root (Supplementary Fig. S2, A and B). The remaining seeds germinated as wild-type-like seedlings (76/228) (Fig. 1E). Notably, all *mrl-1* seedlings developed into normal wild-type-looking plants and successfully completed their life cycle with the production of seeds (Supplementary Fig. S2C).

To investigate where these supernumerary seedlings originate, we compared late embryo stages in *mrl-1* and WT. In WT, the heart shaped embryo is connected by a single file of suspensor cells to the maternal tissue (Fig. 1F). In *mrl-1*, it appears that embryos can develop from suspensors in addition to the primary embryo (Fig. 1G). This phenotype reminded us of the polyembryonic *twn1* mutant, where ectopic embryos also develop from the suspensor (Vernon and Meinke 1994). We therefore crossed both mutants to test for allelism and observed that all F1 seeds germinated single seedlings, indicating *twn1* and *mrl-1* represent mutations in different genes (Supplementary Fig. S2D).

### Development of extra-embryonically derived *mrl-1* embryos

To investigate the origin and development of secondary embryos, we followed their formation from fertilization onward. First, we compared early embryo development in time in WT and *mrl-1* following manual pollination to ensure synchronous fertilization. At 48 hap (hours after pollination), WT embryos were in the dermatogen stage whereas early globular stage embryos occurred at 72 hap (Supplementary Fig. S2, E and G). However, *mrl-1* embryos were in the 2- or 4-cell stage at 48 hap and developed toward the octant stage at 72 hap (Supplementary Fig. S2, F and H). These results indicate a delay in early embryo development in *mrl-1* compared to WT.

**Figure 2.**
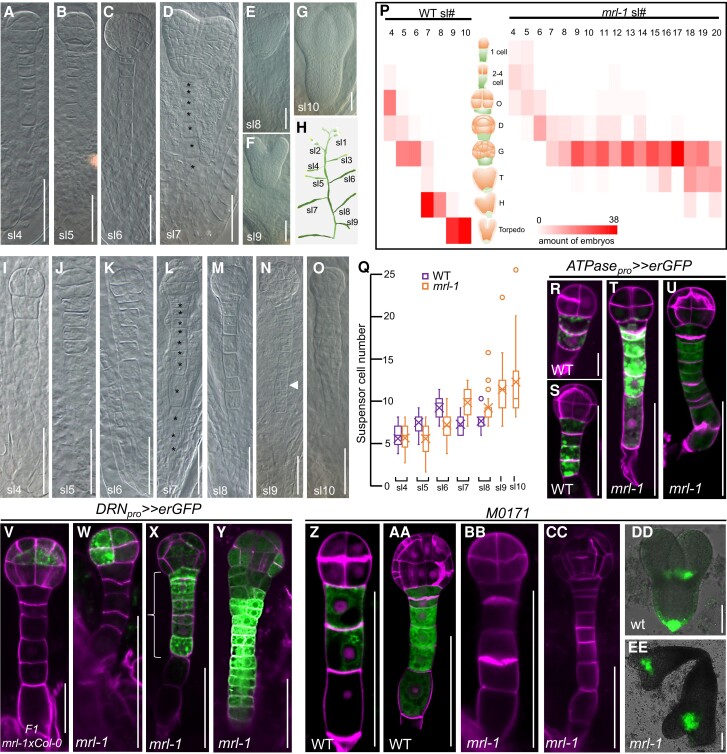
Suspensor derived polyembryony in *mrl-1*. **A to G)** Embryo development in WT corresponding to silique position showing dermatogen (**A**), early globular (**B**), late globular (**C**), early heart (**D**), late heart (**E**), and torpedo stage (**F**, **G**). **H)** Silique position used as reference in microscopy observations. **I to O)** Embryo development in *mrl-1* corresponding to silique position showing 2 to 4 cell (**I**), octant (**J**, **K**), globular (**L** to **N**) and dermatogen stage (**O**). Arrow in (**N**) depicts longitudinal suspensor cell division. Additional ectopic divisions preluding polyembryo development are observed in (**O**). **P**) Embryo proper development in relation to silique position in WT and *mrl-1.* Gradient depicts number of embryos. Cartoons represent stage of embryo development (O: octant; D: dermatogen; G: globular; T: transition; H: heart; and Torpedo stage). **Q**) Suspensor cell number related to silique position. A box and whisker chart shows distribution of data into quartiles, highlighting the mean (cross) and outliers (circle). **R to U**) Representative images of *mrl-1* and WT embryos visualizing *ATPase* promoter activity (R, T octant stage and S, U globular stage) through transactivation of *erGFP* expression. **V to Y**) *DRNpro >> erGFP* expression in globular stage embryo of wild type developing heterozygous *mrl-1^+/−^* (F1 of *mrl-1*(♂)xCol-0, V) and in *mrl-1* at octant (**W**) and globular stage (**X**, **Y**). Bracket indicates ectopic erGFP in suspensor cells of *mrl-1* (**X**). Z-EE. *M0171* enhancer trap expression in WT (**Z**, AA, DD) and *mrl-1* embryos (BB, CC, EE) in octant stage (Z, BB), globular stage (AA, CC), and late heart stage (DD, EE). In R-EE, n (observed embryo numbers) > 5. Scale bar is 50 *μ*m, except R is 10 *μ*m.

Next, we followed embryo development and counted suspensor cell number in relation to silique development after normal self-fertilization. We considered the stage 15 flower as silique 1 (sl1), and subsequently the older flower/silique was referred to as sl2 and so on (Fig. 2H) (Smyth et al. 1990). We mainly observed WT dermatogen stage embryos in sl4. In contrast, *mrl-1* embryo development was delayed and mostly 2- to 4-cell stage embryos occupied sl4 (Fig. 2, A, I, and P; Supplementary Data Set 2). Similarly, while WT sl5 already contained early globular stage embryos, most *mrl-1* embryos were at the octant stage in sl5 (Fig. 2, B, J, and P; Supplementary Data Set 2). Our results were consistent with the manual pollination experiment. Therefore, we continued tracing embryo stages and determined suspensor number in subsequent self-fertilized siliques to illustrate differences in embryo development between WT and *mrl-1*.

Embryo development in WT continued from the dermatogen stage at sl4 toward the torpedo stage within seven consecutive siliques (sl4 to sl10) (Fig. 2, A to G, P; Supplementary Data Set 2). However, *mrl-1* embryo development slowed down toward the globular stage, after which development paused, only to continue much later (Fig. 2, I to O, P; Supplementary Data Set S2). For instance, most WT embryos within sl7 had reached the early heart stage whereas *mrl-1* embryos remained mainly at the globular stage until at least sl17 (Fig. 2P; Supplementary Data Set 2). Nevertheless, fertilized ovules successfully developed mature embryos indicating embryo development catches up during later silique developmental stages.

In WT, the suspensor originates from the large basal cell after the first asymmetric division of the zygote. This basal cell undergoes several transverse divisions to form a mature suspensor consisting of 7 to 12 cells (Fig. 2, A to D, Q) (Yeung and Meinke 1993). However, the *mrl-1* suspensor underwent more transverse divisions (Fig. 2I-N, Q, Supplementary Data Set S3). In addition, we observed longitudinal suspensor cell divisions from sl9 onwards (Fig. 2N), which was never observed in WT. At later stages, additional ectopic suspensor divisions occurred that were the prelude of future embryo-like structures (Fig. 2O).

To investigate whether and when the ectopically dividing suspensor cells in *mrl-1* assumed embryo identity, we checked *DÖRNROSCHEN (DRN)* reporter expression. We transformed *mrl-1* with a binary construct harboring *DRN_pro_ >> erGFP* and crossed a selected T3 homozygous transgenic line with WT to compare expression patterns. In the F1 WT embryo, the *DRN* reporter is specifically expressed in the embryo proper in agreement with its reported expression pattern (Fig. 2V) (Cole et al. 2009). Prior to the globular stage the *DRN* reporter is only expressed in the embryo proper in *mrl-1,* suggesting that the suspensor identity is initially specified in *mrl-1* (Fig. 2W). However, in addition to embryo proper expression, we observed ectopic expression in the transverse dividing suspensor cells around the globular stage (Fig. 2, X and Y). In fact, we could already observe suspensor expression of *DRN* before the suspensor underwent longitudinal cell divisions (Fig. 2X). Next, we examined the integrity of suspensor fate using M0171, an enhancer trap line specifically expressing erGFP in the suspensor of octant-until heart-stage embryos and which later becomes expressed in the cotyledon junction (Fig. 2Z, AA, DD) (Radoeva et al. 2016). However, in *mrl-1* embryos, M0171 expression was absent from the suspensor (*n* > 20), and only the later expression in the cotyledon junction remained (Fig. 2BB, CC, EE). In addition, we tested the *ATPase_pro_* suspensor marker that was reported to be expressed from at least the 4-cell stage onwards (Radoeva et al. 2020). We confirmed its expression in the wild-type and *mrl-1* suspensor (Fig. 2, R to U). The correct setup of the *DRN* marker expression together with the *ATPase_pro_* expression pattern indicate that suspensor fate is initially specified and becomes (partially) lost during early embryogenesis after which ectopic expression of embryo proper fate is followed by suspensor division.

Reprogramming of the suspensor could also be achieved upon inhibition of auxin response in these cells, similarly resulting in aberrant divisions and embryo-specific gene expression (Rademacher et al. 2012). Therefore, we compared auxin response in WT and *mrl-1* embryos, visualized by the *DR5_pro_:nlsVENUS* reporter (Heisler et al. 2005) (Supplementary Fig. S3, A to F). In WT, *DR5* expression shifts from the apical lineage to the hypophysis and uppermost suspensor cells around the young globular stage and, following hypophyseal cell division, to the progenitors of QC and columella stem cells (Friml et al. 2003) (Supplementary Fig. S3, A to C). We could not detect *DR5* reporter expression in the *mrl-1* embryo proper before the globular stage (Supplementary Fig. S3D). Globular and later stage *mrl-1* embryo proper displayed much reduced *DR5* levels, corresponding with delayed development of the embryo proper. Instead, high *DR5* expression was observed in the ectopic transversely divided suspensor cells in *mrl-1* embryos (Supplementary Fig. S3, E and F), cells that later develop embryo structures. This indicates that auxin response in the suspensor is associated with a cell fate transition in *mrl-1*, and suggests a different mechanism of reprogramming compared to that observed upon auxin response inhibition.

Together, these results reveal that *mrl-1* is a polyembryonic mutant in which both embryo proper and suspensor-derived embryos develop.

### Molecular identification of the *MRL* gene

**Figure 3.**
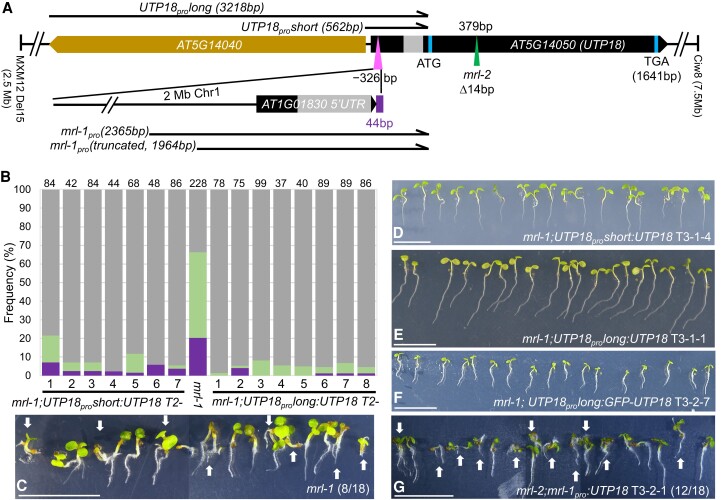
Molecular identification of the *UTP18* gene. **A)** Schematic illustration of the wild type and mutant *UTP18* locus. *UTP18* was mapped on chr 5 between markers MXM12-Del15 and Ciw8. The lines at the site of the triangle 326 bp upstream of the start codon (ATG), indicate the 2 Mb insertion including a 44 bp unknown sequence (bar) in the *UTP18* gene of the *mrl-1* mutant. The *UTP18* transcribed region is represented by the pentagon with an intron indicated in front of the start codon (ATG), a triangle representing the *mrl-2* CRISPR mutation at 379 bp, and TGA representing the stop codon. Numbers are base pairs relative to the start codon of *UTP18.* Introns not indicated in *AT5G14040*. Arrows indicate amplified promoter regions from wild type (*UTP18pro*) and mutant (*mrl-1pro*). **B)** Complementation of *mrl-1* phenotypes by *UTP18* driven by a short (563 bp, *UTP18_pro_short*) or long (3,218 bp, *UTP18_pro_long*) wild-type promoter sequence in segregating T2 lines. Bars are divided in percentage of WT-like seedlings (top section), percentage of polyembryo seedlings (middle), and percentage of polycotyledon seedlings (bottom section). Numbers above columns indicate germinating seeds counted per line. **C)** Germinating *mrl-1* seeds. Arrows indicate multiple seedlings germinating from one seed. Number indicates polyembryonic seeds out of total shown in the image. **D to F)** Complementation of *mrl-1* phenotype by introduction of *UTP18_pro_short:UTP18* (**D**), *UTP18_pro_long:UTP18* (**E**), *UTP18_pro_long:GFP-UTP18* (**F**). All lines depicted are homozygous for the mutation and construct. **G)** Reconstruction of the polyembryo phenotype by introduction of *mrl-1pro:UTP18* into the *mrl-2* knockout. Number indicates polyembryonic seeds out of total shown in the image. Scale bar: 10 mm.

As an initial step to identify the causal mutation we roughly mapped the region conferring the *mrl-1* phenotype on the Arabidopsis genome. We generated a mapping population by crossing the homozygous *mrl-1* mutant in the No-0 background with Arabidopsis accession Landsberg *erecta* (Ler). F2 plants originating from polyembryonic seeds were genotyped using primers based on insertion–deletion polymorphisms (InDels) (Jander et al. 2002). This allowed us to map the *mrl-1* mutation to the short arm of chromosome 5, between markers MXM12-Del15 (2.5 Mb) and Ciw8 (7.5 Mb). We then went ahead to perform whole genome sequencing of the T-DNA free *mrl-1* mutant and its parental HCN line. We identified an approximately 2 Mb duplicated genomic DNA region of chromosome 1 that was reversely inserted into the first exon (−326 bp) of AT5g14050 (Fig. 3A, Supplementary File 1 and S2), which encodes UTP18. The promoter and part of the 5′-UTR of the AT1G01830 gene, encoding an Armadillo repeat superfamily protein, followed by a 44 bp unidentified sequence, was inserted in the 5′-UTR of AT5G14050, 326 bp upstream of its start codon (Fig. 3A). However, this 2 Mb insertion was also located at −246 bp from the start codon of the AT5g14040 gene encoding a MITOCHONDRIAL PHOSPHATE TRANSPORTER 3 (MPT3) in the opposite direction (Fig. 3A).

To determine which of these two genes is causal for the polyembryo phenotype, constructs harboring these genes were transformed into the *mrl-1* mutant and the transgenic progeny was tested for complementation of the polyembryo phenotype. One construct contained AT5G14050 expressed from the 3,218 bp upstream region, named the *UTP18_pro_long* promoter, and expressed both genes. The other construct only contained AT5G14050 expressed from the short 562 bp promoter, hence separating both genes (*UTP18_pro_short*, Fig. 3A). Both constructs completely restored the *mrl-1* phenotypes to WT (Fig. 3, B to E), indicating that AT5G14050 represents the causal gene for *mrl-1*. The AT5G14050 encoded UTP18 protein consists of 537 amino acids and contains four WD40 repeat domains (Supplementary Fig. S4A). Phylogenetic analysis based on amino acid alignment revealed that no UTP18 paralogs exist in the Arabidopsis genome, indicating that UTP18 represents a single-copy gene (Supplementary Fig. S4B). AlphaFold prediction of UTP18 homologs in three model species, Arabidopsis, rice (*Oryza sativa*), *Saccharomyces cerevisiae* and in *Homo sapiens* indicate they have a similar scaffold-like WD40 repeat structure suggesting a conserved function (Supplementary Fig. S4, C to F). UTP18 protein homologs in different species such as *S. cerevisiae*, *Homo sapiens*, and *Drosophila melanogaster* indicate its function as a UTP18 protein involved in nucleolar processing of pre-18S ribosomal RNA (Dragon et al. 2002; Bernstein et al. 2004; Fichelson et al. 2009). UTP18 is a conserved component associated with the U3 small nucleolar ribonucleoprotein (snoRNP) complex which is involved in cleavage of the primary precursor 18S rRNA at the P-site to cut off the 5′ external transcript spacer (ETS) (Fig. 6H) (Venema and Tollervey 1999; Sáez-Vasquez et al. 2004; Sáez-Vásquez and Delseny 2019). Loss-of-function *utp18* alleles are unviable in *Saccharomyces* and *Drosophila* (Vandenbol and Portetelle 1999; Fichelson et al. 2009) which indicates a crucial function for the encoded proteins in development.

### UTP18 localizes to the nucleolus and is ubiquitously expressed

**Figure 4.**
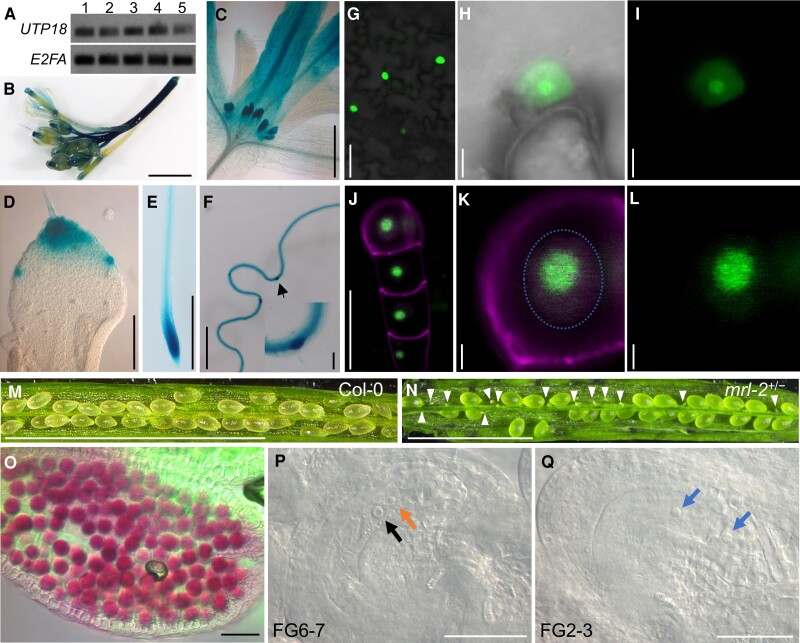
UTP18 localizes to the nucleolus and is required for FM development. **A)***UTP18* mRNA expression relative to the *E2FA* control determined by RT-PCR in Arabidopsis roots (1), stems (2), cauline leaves (3), inflorescences (4) and siliques (5). **B to F)***UTP18* promoter activity examined by GUS staining of *UTP18_pro_long::GUS* transgenic plants showing inflorescence (**B**), 7-d-old shoot (**C**), developing leaf (**D**), seedling root tip (**E**), and older root part developing root primordia (**F**). Arrow and inset indicate lateral root primordium (**F**). **G to I**) Transient expression of *35S_pro_:GFP-UTP18* in *N. benthamiana* leaf cells showing nuclear accumulation with highest levels in nucleolus. Overview (**G**) and single nucleus focus with overlay of bright field and fluorescence channel (**H**) and fluorescence channel only (**I**). (**H**) and (**I**) are representative of >10 imaged cells. **J to L**) Transgenic embryo expressing *UTP18_pro_long:GFP-UTP18* showing accumulation in nucleolus. Overview (**G**) and single nucleus focus (dashed line) in embryo proper with overlay (**H**) and fluorescence channel only (**I**). (**J) to (L**) is representative of >5 imaged embryos. **M to N**) Dissected siliques of WT showing normal ovules (**M**) compared to *mrl-2*^*+/*−^ silique segregating aborted ovules (**N**, arrowheads). **O**) Pollen vitality assay by Alexander staining of *mrl-2*^+/−^ stamen. Staining indicates viable pollen. **P, Q**) Asynchronous ovule development in *mrl-2*^+/−^ showing a FG6-7 stage ovule (**P**) and a FG2-3 stage ovule (**Q**) both from a stage 4 silique. Left arrow indicates a bigger secondary nucleus (fused polar nuclei) and a smaller nucleus (right arrow) in the FG6 stage (**P**). Arrows indicate two equal size nuclei in the FG2-3 stage (**Q**). Scale bars in B, E, F, M, N is 5 mm, C, D, G is 100 *μ*m, J, O, P, Q and F inset is 50 *μ*m, H, I, K, L is 5 *μ*m.

To determine in which tissues the *UTP18* gene is expressed, we performed RT-PCR with *UTP18*-specific primers, which resulted in the amplification of a single fragment with expected size from cDNA of roots, stems, cauline leaves, inflorescences, and siliques (Fig. 4A). Public transcriptome data also indicate ubiquitous presence of *UTP18* mRNA albeit with higher levels in dividing tissues (Supplementary Fig. S5A). We generated reporter lines by transcriptionally fusing the 3,218 bp long *UTP18* promoter region to β-Glucuronidase (GUS), creating *UTP18_pro_long:GUS*, and similarly observed expression in all tissues of the plant (Fig. 4, B to F). Strongest GUS staining was observed in actively proliferating tissues such as leaf tip, root tip, and lateral root primordia (Fig. 4, B to F), consistent with a protein function in regions of high demand for ribosome biogenesis.

UTP18 is reported to process the pre-18S rRNA in the nucleolus of yeast cells (Dragon et al. 2002). To determine the subcellular localization of UTP18 in Arabidopsis, we generated a construct expressing a GFP-UTP18 protein fusion under control of the strong and constitutive Cauliflower Mosaic Virus 35S promoter. This construct was transiently expressed in *Nicotiana benthamiana* leaves by Agrobacterium-mediated infiltration, and protein accumulation was examined by confocal scanning laser microscopy. The GFP-UTP18 fusion protein localized to the nucleus with the highest concentration in the nucleolus (Fig. 4, G to I). To confirm the nucleolar accumulation in Arabidopsis, we transformed *mrl-1* plants with a construct harboring *UTP18_pro_long:GFP-UTP18*, and that was able to complement the polyembryo phenotype (Fig. 3F). We observed the fluorescent GFP signal predominantly in the nucleolus (Fig. 4, J to L).

### UTP18 is essential for female gametophyte development

To investigate the incomplete penetrance of the polyembryo phenotype in *mrl-1* and the corresponding mutation in the 5′-UTR of the *UTP18* gene, we generated additional alleles in the coding sequence using CRISPR/Cas9 in the Arabidopsis ecotype Col-0 background. Two alleles were pursued: one with a 14 bp deletion 379 bp downstream of the start codon thereby introducing a premature stop codon (*mrl-2*, Fig. 3A), and one with a 12 bp homozygous deletion 380 bp downstream of the start codon (*mrl-3*). The *mrl-3* allele did not display an obvious phenotype indicating that a four amino acid deletion at this position in the protein can sustain its function. For the *mrl-2* allele we could not recover homozygous mutant offspring (*n* > 100), which corresponded well to approximately one-third of ovules being aborted in siliques of heterozygous *mrl-2*^+/−^ plants (Fig. 4, M and N; Supplementary Data Set 4).

To determine the origin of the observed homozygous lethality we performed reciprocal crosses between *mrl-2^+/−^* and Col-0 plants. Fertilization of *mrl-2^+/−^* flowers with Col-0 pollen resulted in WT F1 progeny only, suggesting that the mutation influences female gametophyte development thereby preventing fertilization. Vice versa, fertilizing Col-0 flowers with pollen of *mrl-2^+/−^* plants and genotyping of eight F1 plants gave only one heterozygous plant. This skewed segregation ratio suggested that the *mrl-2* mutation also influences the male gametophyte. To assess pollen vitality, we compared pollen from stamens of two heterozygous *mrl-2^+/−^* plants with those from WT using a simplified Alexander’s staining method (Peterson et al. 2010). This experiment showed that *mrl-2^+/−^* plants produced pollen that all appear normal (Fig. 4O).

Next, we examined formation of the female gametophyte (FG) whose development was ordered in stages FG1 to FG8 (Christensen et al. 1998; Drews and Koltunow 2011). Following megaspore meiosis, the remaining functional megaspore undergoes nuclear division, nuclear migration and cellularization, eventually forming a seven-celled mature FG (Drews et al. 1998; Drews and Koltunow 2011). We examined FG stages across five sequential siliques (sl1 to sl5) in *mrl-2^+/−^* and observed that ovule development was asynchronous in *mrl-2*^+/−^ compared to in WT (Table 1; Supplementary Fig. S6). For example, of the 35 ovules examined from the sl4 stage, 15 ovules were at FG6 (Fig. 4P), 13 ovules were at FG2-3 (Fig. 4Q), whereas FG4 and FG5 stage ovules were lacking (Table 1). In contrast, most ovules in sl4 from WT were at FG6 stage (Table 1). We conclude that UTP18 is essential for female gametogenesis, thereby explaining the absence of homozygous mutant progeny.

**Table 1.** Developmental stages of FG in WT and *mrl-2*^+/−^ flowers

	Pistil number	FG0	FG1	FG2-3	FG4	FG5	FG6	FG7-8	Zygote
Col-0	1sl	32	8						
	2sl	3	12	17	15				
	3sl			8	14	28	2		
	4sl				4	4	36	5	
	5sl							8	43
*mrl-2/+*	1sl	1	21	13	5				
	2sl		16	9	3	4	4		
	3sl		14	14	2	1	10		
	4sl		2	13			15	5	
	5sl			12	3				19

FG stages were compared WT according to Christensen et al. (1998).

### Altered embryonic *UTP18* expression causes the polyembryo phenotype

Given that the homozygous *mrl-2* mutant cannot be obtained, the polyembryo phenotype in *mrl-1* may be due to altered gene expression because of the 2 Mb DNA insertion whereby the promoter and part of the 5′-UTR of the AT1G01830 gene now controls *UTP18* expression (Fig. 3A). To investigate the role of the mutant promoter in conferring the polyembryo phenotype, we first compared public expression data available for AT1G01830 and *UTP18* (AT5G14050). It appears that AT1G01830 is generally expressed at lower levels during Arabidopsis development, particularly during embryogenesis (Supplementary Fig. S5, A and B).

To confirm that altered regulation of *UTP18* is responsible for the polyembryo phenotype, we first transformed heterozygous *mrl-2*^+/−^ plants with the WT *UTP18* coding sequence under control of the 2365bp *mrl-1* mutant promoter (*mrl-1_pro_:UTP18*) and genotyped for homozygous *mrl-2* mutant progeny in the next generation. We identified two double homozygous *mrl-2; mrl-1_pro_:MRL* T3 lines with polyembryo and polycotyledon phenotypes similar to *mrl-1* (Fig. 3G; Supplementary Fig. S2, I to K). Interestingly, the penetrance of polyembryonic seeds (63% and 54%) was higher in the double homozygous lines than in *mrl-1* (45%) (Supplementary Data Set 1).

We then compared activities of the wild-type *UTP18_pro_long* and mutant *mrl-1_pro_* promoters driving a *3xVENUSnls* in three independent transgenic lines generated for each construct in the Col-0 background. In early embryonic stages, we observed much lower VENUS fluorescence in both suspensor and embryo proper for the *mrl-1_pro_:3xVENUSnls* construct compared to that of the wild-type promoter construct *UTP18_pro_long:3xVENUSnls,* using the same confocal settings (Supplementary Fig. S7, A and C). By the heart stage of embryogenesis, the VENUS signal could be detected at around the same level for both promoter constructs, albeit with a different, but consistent, pattern of expression depending on the genetic background (Supplementary Fig. S7, B and D).

**Figure 5.**
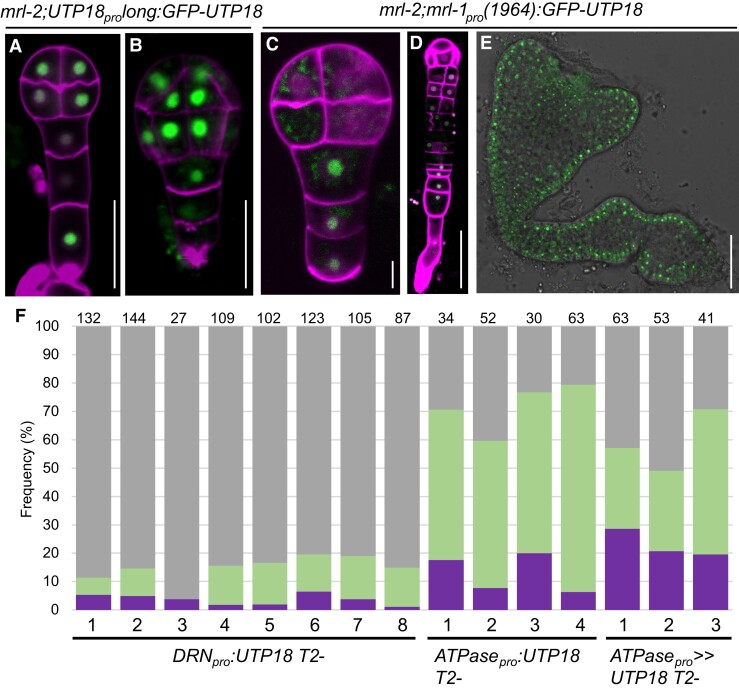
Altered *UTP18 e*xpression pattern is causal to the polyembryo phenotype. **A, B**) Complementation of the loss of function *mrl-2* by expression of *UTP18_pro_long:GFP-UTP18* exemplified by formation of WT looking octant (**A**) and dermatogen (**B**) stage embryos. **C to E**) Expression of *mrl-1_pro_(1964):GFP-UTP18* in the loss of function *mrl-2* reconstitutes the *mrl-1* polyembryo phenotype. Misexpression of UTP18 in octant (**C**) stage embryo is associated with delayed embryo proper development and suspensor reprogramming (**D**). Recovery of UTP18 expression in the primary embryo at later developmental stages is associated with polyembryo formation (**E**). **F)** Phenotypes of *mrl-1* are complemented by embryo proper expression of *DRN_pro_:UTP18,* but not by suspensor specific *ATPase_pro_:UTP18* and *ATPase_pro_ >> UTP18* expression. Numbers above columns indicate germinating seeds counted per line. Phenotypes are determined in transgenic T2 lines. Green represents germinating twin and triple seedlings, purple represents polycotyledon seedlings, and grey represents WT-looking seedlings. In A-E, n (observed embryo numbers) > 5. Scalebars are 50 *µ*m, except in C 5 *µ*m.

Next, we aimed to compare the protein abundance of UTP18 during WT and polyembryo development. Using the same strategy as above we generated transgenic lines in the homozygous *mrl-2* background expressing *GFP-UTP18* from the *UTP18_pro_long* promoter and a slightly truncated *mrl-1_pro_* (1,964 bp) promoter. The introduction of the *UTP18_pro_long:GFP-MRL* construct fully complemented the seed abortion phenotype of *mrl-2* (compare Fig. 4N and Supplementary Fig. S8A). Correspondingly, GFP signal was observed in all embryonic cells at early embryonic stages (Fig. 5, A and B). To examine UTP18 abundance during polyembryony we used the reconstituted *mrl-1* phenotype displayed by the *mrl-2*; *mrl-1_pro_(1964):GFP-UTP18* genotype. We pursued a T1 line for which ovules displayed asynchronous development within a silique (Supplementary Fig. S8B). These delayed ovules resemble the delay in ovule development displayed by *mrl-1* when compared to the No-0 WT background in subsequent siliques (Supplementary Fig. S8, D to K). Indeed, we found suspensor-derived embryo structures formed in these delayed ovules (Fig. 5E), and additional seedlings germinated from single seeds (Supplementary Fig. S8C). Based on this, we considered the GFP signal to represent the true UTP18 protein abundance in the *mrl-1* polyembryonic mutant. Interestingly, GFP-UTP18 was absent from the embryo proper during early embryo stages (Fig. 5, C and D). This altered expression during the early embryonic stages corresponds with the observed ectopic divisions in the suspensor in later stages (Fig. 5D) that served as a prelude to suspensor-derived embryogenesis. In these later embryo stages, both embryo proper and suspensor (which includes suspensor-derived embryos) expressed GFP-UTP18 protein (Fig. 5E).

**Figure 6.**
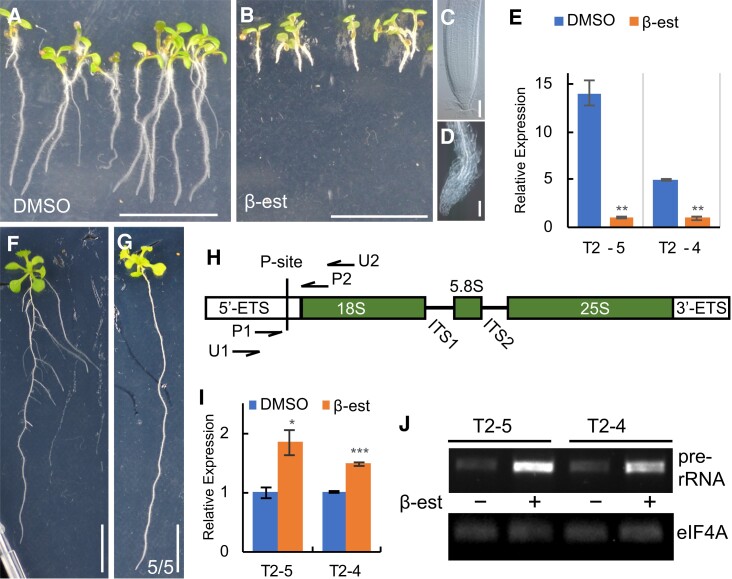
Downregulation of *UTP18* prevents growth and processing of pre-18S rRNA **A to D**) Transgenic T2-5 seedlings of *G1090_pro_ >> zCas9i-AtU6_26_pro_:UTP18sgRNA1&2* germinated and induced for 4 d on DMSO (**A**, **C**) or 10 *μ*M β-estradiol (**B**, **D**). **E)***UTP18* mRNA levels determined by RT-qPCR in two T2 lines germinated on 10 *μ*M β-estradiol relative to DMSO. **F, G**) Representative 15-d-old Col-0 seedling (**F**) and a transgenic T2-1 *PLT7_pro_:zCas9i-AtU6_26_pro_:UTP18sgRNA1&2&3* seedling lacking lateral roots (representative of five seedlings without lateral roots) (**G**). **H**) Schematic representation of the pre-rRNA transcript in Arabidopsis showing the 18S, 5.8S and 25S rRNAs separated by internal transcript spacers (ITS) and flanked on the extremities by a 5′ and 3′ external transcript spacer (ETS). The P site is the primary cleavage site of the pre-rRNA. Primers U1, U2, P1, and P2 are used in the RT-PCR experiments. **I)** RT-qPCR showing the amount of 18S pre-rRNA in transgenic callus derived from T2-4 and T2-5 seedlings expressing *G1090_pro_ >> zCas9i-AtU6_26_pro_:UTP18sgRNA1&2* induced with β-estradiol or DMSO. **J)** RT-PCR showing increased levels of unprocessed 18S pre-rRNA in transgenic T2-4 and T2-5 seedling roots expressing *G1090_pro_ >> zCas9i-AtU6_26_pro_:UTP18sgRNA1&2* induced with β-estradiol (+) or DMSO (−). Bottom image indicates *EIF4A* mRNA levels showing that equal amounts RNA template were used. Scale bar in A, B, F, G is 10 mm; scale bar in C, D is 50 *μ*m. Bars and asterisks in E, I, (*); (**); (***) represent Sd and significant difference at *P* < 0.05; 0.01; 0.001, two-tailed *t*-test.

Together these results indicate that the abundance of UTP18 is altered predominantly during early embryogenesis leading to halted embryo proper development and ectopic suspensor-derived embryo formation.

### UTP18 functions noncell autonomously from the embryo proper to maintain suspensor identity

The embryonic transformation of the suspensor, as observed in *twn1* mutants, was proposed to be due to loss of communication between embryo proper and suspensor (Vernon and Meinke 1994; Schwartz et al. 1997). Here we observe very similar phenotypes for *mrl-1*, and our expression and complementation experiments above suggest an important role for sufficient and local expression of UTP18 during development. Therefore, we tested where *UTP18* expression is limiting for maintaining embryo homeostasis by complementation experiments utilizing specific promoters to drive *UTP18* in the *mrl-1* background.

We used the *DRN* promoter to drive *UTP18* expression in the apical cell lineage after asymmetric division of the *mrl-1* zygote. *DRN* reporter expression was limited to the embryo proper in *mrl-1* early embryo development before its ectopic expression from the globular stage onward (Fig. 2, W to Y), allowing its use in complementation experiments. Next, we introduced *DRN_pro_:UTP18* into the *mrl-1* mutant and observed in 8 out of 10 T2 lines a substantial reduced penetrance of polyembryo and polycotyledon phenotypes (Fig. 5F; Supplementary Data Set 5). Of these, we generated two lines homozygous for the transgene (T3-1-1 and T3-2-1) that showed 100% complementation (Supplementary Fig. S9A).

To drive *UTP18* expression in the suspensor we used the *ATPase* promoter (Radoeva et al. 2020), that is active in the *mrl-1* suspensor (Fig. 2, T and U). To test whether *UTP18* expression in the suspensor can rescue the polyembryo phenotype of *mrl-1*, we generated plants with the *ATPase_pro_ >> UTP18* or *ATPase_pro_:UTP18* transgene. None of the T2 transgenic lines nor a next generation homozygous T3 line showed reduced penetrance of polyembryony or polycotyledony (Fig. 5F;Supplementary Data Set 5, Supplementary Fig. S9B).

Taken together, our findings suggest that sufficient levels of *UTP18* expression are required in and during early embryo proper development to nonautonomously prevent the formation of suspensor derived embryos that are causal to the polyembryo phenotype of *mrl-1.*

### UTP18 is involved in processing of pre-18S rRNA

In plants, the 45S rDNA encodes the 18S, 5.8S, and 25S rRNAs on a single transcription unit, and exists in hundreds of copies in the genome. The resulting primary transcripts, the pre-rRNAs, harbor the individual rRNAs. These are separated by internal transcript spacers (ITS) and the outer boundaries are determined by the ETS. The spacers are subsequently removed in a complex maturation process inside the nucleolus through transient interaction with a large so-called small subunit processome or U3 snoRNP complex that also mediates the early endonucleolytic cut in the 5′-ETS upstream from the 18S rRNA (Fig. 6H) (reviewed in Tomecki et al. 2017; Sáez-Vásquez and Delseny 2019). To investigate whether the molecular function of UTP18 is consistent with being the Arabidopsis UTP18 homolog and thereby part of this U3 snoRNP complex, we chose a conditional CRISPR approach because of the lethal *mrl-2* knock-out phenotype. We generated two constructs, one for induced constitutive and one for tissue-specific CRISPR/Cas9, that were introduced into Arabidopsis Col-0. The induced constitutive construct *G1090_pro_ >> zCas9i-AtU6_26_pro_:UTP18sgRNA1&2*, expressed the synthetic XVE-encoded synthetic transcription factor (Zuo et al. 2000) to transactivate *lexA* promoter driven *zCas9i* (Grützner et al. 2021) upon addition of β-estradiol, and also expressed the single guide RNAs targeting the *UTP18* CDS. The tissue specific construct *PLT7_pro_:zCas9i-AtU6_26_pro_:UTP18sgRNA1&2&3,* expressed the *zCas9i* from the 1.5 kb lateral root primordium specific *PLT7* promoter (Du and Scheres 2017).

To test the efficacy of the constitutive CRISPR/Cas9-induced mutations and the associated post-embryonic phenotypes, we germinated transgenic seeds or transferred seedlings of two inducible T2 lines (T2-4 and T2-5) onto medium containing 10 *μ*M β-estradiol and compared these to a DMSO mock control. Seedlings stop growing upon Cas9 induction as visualized by loss of meristem activity 4 days post-induction (dpi), in contrast to normal-growing mock-treated control roots (Fig. 6, A to D). *UTP18* expression was decreased in seedlings germinated on Cas9 induction media compared to in control seedlings (Fig. 6E), possibly due to the loss of dividing tissues that require high UTP18 levels. For the lateral root specific CRISPR/zCas9i T2 seedlings, we observed an absence of emerging lateral roots compared to the multiple lateral roots emerging from WT primary roots of the same age (Fig. 6, F and G). These results indicate that the conditional CRISPR/zCas9i targeting of *UTP18* is effective, and that UTP18 is essential for post-embryonic (root) growth.

Previously, SLOW WALKER 1 (SWA1), a UTP15 homolog and one of the components of the U3 snoRNP, was shown to be involved in the endonucleolytic cleavage at the P-site of the 5′-ETS from the pre-18S rRNA. This was demonstrated using callus derived from roots (Shi et al. 2005). To investigate a similar function for Arabidopsis UTP18 as a yeast UTP18 homolog, we used the conditional mutant strategy to induce loss of gene function and subsequently test pre-rRNA cleavage in the affected tissues. Therefore, we generated callus from 9-d old transgenic roots of the same inducible constitutive CRISPR/Cas9 T2 lines used above. After culturing the roots on callus-inducing medium for 2 d, *Cas9* was induced for the final 9 d with 10 *μ*M β-estradiol or with DMSO as mock treatment. Total RNA was isolated from these 11-d-old calli and RT-qPCR was performed with primers P1 and P2 that flank the P-site (Fig. 6H). The results show that the amount of unprocessed pre-18S rRNA increased almost 2-fold and 1.5-fold in callus derived from CRISPR/Cas9-induced lines T2-5 and T2-4, respectively, compared to the control (Fig. 6I). We also tested pre-18S rRNA cleavage in 14-d-old T2-4 and T2-5 seedling roots that were induced for 9 d upon transfer to 10 *μ*M β-estradiol or DMSO mock medium (Supplementary Fig. S10, A to D). RT-PCR was performed using root total RNA with primers U1 and U2 as described in Shi et al. (2005). The results similarly show that the amount of uncleaved pre-18S rRNA increased in CRISPR/Cas9-induced roots compared to control roots (Fig. 6J). Taken together, these data indicate that the UTP18 protein is required for the primary cleavage of P-site during the processing of the pre-18S rRNA, consistent with its annotation as a homolog of yeast UTP18.

## Discussion

In this study, we identified *mrl-1* as a polyembryonic mutant. The *mrl-1* mutant displays a high frequency of seeds containing two or more embryos. All viable seedlings gave rise to fertile plants that successfully complete their life cycle with the production of seeds. The supernumerary seedlings in *mrl-1* seeds originate from the suspensor, which normally degenerates at late embryogenesis. The mutant phenotype is caused by an Agrobacterium transformation-induced duplication of a 2 Mb piece of DNA from Chr1 in the 5′ untranslated region of a *UTP18* homolog. The high degree of sequence identity between this Arabidopsis UTP18 and its homologs in other species, together with our experimental data indicate that it is an essential component of U3 snoRNP involved in 18S preribosomal RNA cleavage. The insertion mutation in *mrl-1* generally lowered the level of expression of the *UTP18* gene, especially in dividing tissues and during early embryo proper development. Our results provide detailed molecular evidence for the hypothesis that normal progression of embryo proper development is required to remain suspensor quiescence.

In addition to UTP18, several other members of the U3 snoRNP have been identified as mutants with similar developmental defects. For example, the defective female gametogenesis phenotype causing lethality in the knockout *mrl-*2 allele, is similar to the mutant phenotype of *swa1* (Shi et al. 2005). *SWA1* encodes a homolog of the yeast UTP15 within the U3 snoRNP complex. However, the seed abortion segregation ratio in the heterozygous *mrl-2^+/−^* plant does not follow Mendelian genetics, which suggests UTP18 is also required during fertilization. The *TORMOZ* (*TOZ*) and *POPCORN* (*PCN)* genes encode homologs of the yeast UTP13 and UTP4 U3 snoRNP members, respectively (Griffith et al. 2007; Xiang et al. 2011). The *toz* mutant did not appear to be a null allele and it displayed only a slight effect on rRNA processing in ex-plant cultured embryos. The mutant showed aberrant embryonic cell division planes and arrested development before the globular stage. The *pcn* mutant showed cotyledon phenotypes, delayed embryo proper development and longitudinal suspensor divisions similar to *mrl-1*. Auxin distribution and response were altered in *pcn*, exemplified by ectopic DR5 in suspensor cells, reminiscent of *mrl-1* mutant embryos, although the suspensor successfully regenerates embryo(s) in *mrl-1* as opposed to its developmental arrest in *pcn*. Together, these mutants indicate that phenotypic severity depends on the allele and corresponding protein inside the U3 snoRNP complex, with arrest in female gametogenesis as the most severe phenotype. These effects may be attributed to defective 18S rRNA processing resulting in a general arrest of growth. However, the molecular effects of mutations in the pre-rRNA processing may be diverse, ranging from apoptosis to reduced translation to changes in the translatome (reviewed in Aubert et al. 2018).

Based on the observed phenotypes in *twn* mutants and the ablation studies, the prevalent hypothesis stated that the presence of the embryo proper suppresses secondary embryo development from the suspensor (Vernon and Meinke 1994; Gooh et al. 2015; Liu et al. 2015). In our *mrl* mutant reconstruction line, whereby we introduced a *GFP-UTP18* fusion expressed from the *mrl-1_pro_* promoter in the *mrl-2* knockout that successfully reproduced the polyembryo phenotype, we observed GFP-UTP18 fluorescence only in the suspensor at early embryo stages. This contrasts with MRL expression in both embryo proper and suspensor in WT. In addition, expression of *UTP18* from the *DRN* embryo proper promoter complemented the polyembryo phenotype whereas the suspensor (*ATPase_pro_*) driven *UTP18* did not. Together with the delayed embryo proper development, these data indicate that UTP18 protein abundance in the embryo proper falls below the level to sustain embryo proper vitality. As a consequence, the suspensor initiates secondary embryogenesis. However, upon reaching the transition stage, GFP-UTP18 was again detected in the embryo proper as well as in suspensor-derived embryo, explaining the survival of the original embryo in addition to the formation of ectopic embryos. Together, these results merge into a model on the requirement for UTP18 during early embryogenesis.

Previous laser ablation studies showed that damage to the apical cell that is formed after the asymmetric division of the zygote can induce basal cell reprogramming (Gooh et al. 2015; Liu et al. 2015). Gooh et al. observed cell fate conversion of the upper one or two suspensor cells by subsequent ectopic transverse suspensor cell division followed by loss of suspensor properties (*WOX8* expression) and gain of embryonic properties (*DRN* expression), prior to the first longitudinal embryonic cell division (Gooh et al. 2015). In their experiments, Liu et al. observed *DR5* reporter accumulation in the free end of the suspensor (topmost one or two cells) after ablation, and this cell further developed into an embryo structure. The authors hypothesized that without the embryo proper, auxin transport from the suspensor to the embryo proper was blocked in the topmost suspensor cell, leading to reprogramming of cell fate (Liu et al. 2015). Similar to the above observations, the *mrl-1* single file suspensor cells first undergo transverse divisions. Suspensor cells in *mrl-1* express the *ATPase_pro_* marker but failed initiation of M0171 expression at the octant stage supporting the notion of reprogramming suspensor fate toward embryonic competence. Subsequently these reprogramming suspensor cells display high *DR5* accumulation at the globular stage. Importantly, the embryo proper marker *DRN* is expressed before longitudinal divisions leading to embryo development occur, suggesting that reprogramming is the driver for the divisions leading to embryo formation. In agreement, induction of suspensor cell division through ectopic *bHLH49* expression was not sufficient to induce embryogenesis (Radoeva et al. 2019), likely due to the absence of reprogramming.

The auxin response maximum in the ectopic suspensor cells observed in the ablation studies and in the *mrl-1* suspensor corresponds with suspensor-derived embryo development but may not be the trigger of the suspensor reprograming. This is consistent with the formation of twin embryos upon auxin response inhibition (Rademacher et al. 2012). Using a set of ubiquitous and suspensor drivers to express a nondegradable bodenlos (bdl) protein, excessive aberrant divisions were observed in the suspensor. However, only the constitutive *RPS5A* promoter-driven *bdl* delivered occasional true twin embryos, which corresponded with the ectopic expression of embryonic markers SHOOT MERISTEMLESS (*STM*) and WUSCHEL (*WUS*). Together with evidence on additional ARF and IAA factors in suspensor development, it was concluded that auxin response is required to maintain suspensor cell identity (Rademacher et al. 2012). Suspensor reprogramming was also observed upon ectopic expression of embryo-related transcription factors RWP-RK DOMAIN CONTAINING 1 (RKD1), RKD4, and WUS (Radoeva et al. 2020). Here, suspensor identity is lost, which is followed by ectopic suspensor cell division and subsequent *DRN* embryo proper marker expression. This process shared similarity to somatic embryogenesis, and may resemble a regeneration-like mechanism whereby cells first dedifferentiate and proliferate followed by re-specification and expression of embryonic genes leading to the formation of somatic embryos (Ikeda-Iwai et al. 2003; Horstman et al. 2017; Radoeva et al. 2020). Together, our results suggest a case where suspensor-derived polyembryony as observed in *mrl-1* follows a reprogramming mechanism similar to that observed in the ablation studies and different to the auxin response inhibition and ectopic transcription factor studies.

Embryo ablation studies indicated that the suspensor has the potential to form an embryo up to the globular stage only (Gooh et al. 2015; Liu et al. 2015). Taking advantage of the high penetrance of the *mrl-1* phenotype, we show that delayed development of the embryo proper prior to the transition stage was accompanied by suspensor-derived secondary embryo formation. These observations, combined with the presence of the *ATPase_pro_* expression and failure of M0171 marker initiation at the octant stage in the *mrl-1* mutant places the timing of suspensor reprogramming before or around the octant stage of embryo development. Indeed, *DRN* marker expression is clearly observed in suspensor cells in globular stage embryos.

The *mrl-1* and *twn1* mutants may represent a distinct clade of polyembryonic mutants because suspensor-derived embryogenesis does not depend on injury to the embryo proper. Specifically, in *mrl-1*, embryo development is asynchronous where approximately half of the delayed transition stage embryos exhibit suspensor-derived embryogenesis while the other half continues the typical normal embryo development including suspensor. Nevertheless, embryos from wild-type plants do not develop suspensor-derived embryos at this stage in development, suggesting crucial signaling between embryo and maternal tissues and/or between embryo proper and suspensor. The synchronization between embryo proper and suspensor development appears disturbed with the delay imposed specifically on embryo proper development by the *mrl-1* mutation. The molecular function of the causal mutated gene in *mrl-1* suggests that any factor causing a specific delay only in embryo proper development prior to the globular stage will result in suspensor reprogramming and subsequent embryo development. Such a scenario supports active signaling between the embryo proper and suspensor. If auxin is not the cue for reprogramming the suspensor the question remains what is the role and nature of this signaling? An analogy may be drawn to the opposing (nonautonomous) signaling in the maternal and embryonic control over embryonic root development mediated by a complex interplay of *WIP* gene expression (Du et al. 2022). Crosstalk between these genes may represent a module to deal with local conditions and shift resources toward defense or reproduction (Du et al. 2022; Wittmer and Heidstra 2022). Interestingly, WIPs act through so-called hub proteins able to interact with various transcription factors and mutation of these hub genes alleviated the growth defects in *wip* mutants (Du et al. 2022). In a similar way, the *mrl-1* mutant phenotype may now be applied to screen for signaling components between embryo proper and suspensor, e.g. utilizing a suppressor screening in *mrl-1* background for absence of suspensor derived embryo development or by single-cell RNA sequencing.

In conclusion, our study shows that embryo proper-mediated inhibition of suspensor reprogramming ensures the general one seed–one embryo rule. Our results suggest an active signaling of developmental progression between embryo proper and suspensor to sustain the development of a single dominant embryo. Because of its high penetrance of polyembryony, *mrl* mutants provide an excellent tool for unraveling communication mechanisms between the embryo proper and suspensor.

### Materials and methods

#### Plant material and growth conditions

Arabidopsis (*A. thaliana*) seeds were gas sterilized for 2 h as described in Lindsey et al. (2017), resuspended in sterile 0.1% (w/v) agarose and stratified at 4 °C for at least 2 d. Seeds were subsequently plated on 0.5× Murashige and Skoog medium (MS including vitamins), supplemented with 0.8% (w/v) plant agar, 1% (w/v) sucrose, and 0.5 g/L MES monohydrate pH5.8 (all from Duchefa Biochemie). Plates were positioned near vertical and seedlings were grown at 22 °C with 16 h light and 8 h dark cycle with a light intensity of ∼85 *µ*mol m^−2^ s^−1^ from fluorescent lighting. Seedlings were transferred to soil and grown in a growth chamber under the same 22 °C and long-day conditions but with a light intensity of 120 to 140 *µ*mol m^−2^ s^−1^ from LED lights (Lumeco).

Unless otherwise specified, we used *A. thaliana* ecotype No-0, Col-0 and Ler backgrounds as plant material. The mutant *mrl-1* originated from an *A. tumefaciens* (Agrobacterium)-mediated transformation of the *pCB1* construct (Heidstra et al. 2004) into ecotype No-0, carrying *HSP18.2_pro_:CRE* (*HCN*) (Sieburth et al. 1998) whereby a single T2 line produced seeds germinating multiple seedlings (Fig. 1A). Subsequent rounds of selfing resulted in segregation of the polyembryo phenotype from the *pCB1* T-DNA insertion (Supplementary Fig. S1, C and D). M0171 was described in Radoeva et al. (2016) and *twn-1* seeds originate from Vernon and Meinke (1994).

### Map-based cloning

Seedlings displaying the *mrl-1* phenotype in the F2 population resulting from a cross between *mrl-1* and Landsberg *erecta* (Ler) were used as the mapping population. Using primers based on InDels between Col 0 and Ler (Jander et al. 2002), we were able to roughly map the mutation in between markers MXM12-Del15 (MXM12-Del15F: 5′-GCCAATTTCAACAACGAAGG, MXM12-Del15R: 5′-ATTCGCCGTCGGAATTATCT) and ciw8 (CIW8-F: 5′-TAGTGAAACCTTTCTCAGAT; CIW8-R: 5′-TTATGTTTTCTTCAATCAGTT) in a population of 28 plants selected for the *mrl-1* phenotype out of a total of 60 F2 progeny. Subsequent fine mapping using additional *mrl-1* type seedlings segregating from a total of 800 F2 progeny did not result in getting closer to the mutation. DNA from *mrl-1*, Ler, and the F2 population seedlings was isolated from leaves using the CTAB method (Lukowitz et al. 1996). PCR was carried out in a total volume of 20 *μ*L, containing 0.5 *μ*L of 5U/*μ*L home-made Taq polymerase, 0.5 *μ*L of 10 mmol l^−1^ forward and reverse primers, 2.5 *μ*L of 10× PCR buffer, 0.5 *µ*L of 250 ng/*µ*L DNA, and 16 *µ*L of Milli Q water. All PCR products were detected on a 1% (w/v) agarose gel using electrophoresis.

Whole genome sequencing with paired-end reads was performed on genomic DNA isolated from the T-DNA free *mrl-1* mutant and its parental HCN line on an Illumina HiSeq2000 sequencing machine (BioProject ID PRJNA1009023). Reads were mapped on the TAIR10 reference genome, with the *pCB1* T-DNA and *HCN* sequences added as an additional sequence, using bwa mem (bwa Version: 0.7.12-r1039), and a BAM alignment file was produced with samtools Version: 0.1.19-44428cd. The BAM file was realigned using GATK, and GATK was used to call SNPs and indels. For structural variant analysis, the tool Delly was used (Rausch et al. 2012). Since neither analysis delivered leads as to the identity of the mutation, we resorted to visual inspection of the isolated genomic region in between the markers used for mapping using the Integrative Genome Browser (IGV) (Robinson et al. 2011) to identify the *mrl-1* mutation.

### Construct design, cloning, and plant transformation

All plasmids were constructed by Golden Gate cloning using the MoClo Toolkit and Plant parts (Addgene Kits #1000000044 and #1000000047) (Engler et al. 2014) or gateway cloning (Invitrogen) unless otherwise specified (Supplementary Data Sets 6 and 7). Transgenic plants were generated by Agrobacterium-mediated transformation by means of the floral dip method (Clough and Bent 1998).

To generate *DRN_pro_ >> erGFP* and *ATPase_pro_ >> erGFP* reporter lines, we amplified promoter sequences up to 4,771 bp for *DRN* and 982 bp for *ATPase*, all located upstream of their respective ATG start codons. For the *DRN* reporter, the *pNOS-BAR* selection cassette, *DRN_pro_:GAL4VP16*, and *UAS_pro_:erGFP* were then assembled into the destination vector *pICSL4723* using BpiI Golden Gate assembly. For the *ATPase* reporter construct, we used the FAST-Red selection cassette instead of Basta. The reporter plasmids were transformed into *mrl-1*, and we confirmed that the reporter signals were consistent across three independent homozygous lines.

We constructed *UTP18_pro_long:GUS* by gateway cloning the 3,218 bp *UTP18* promoter region (Fig. 3A) in front of GUS in the *pGII0229R4R3* pGreen vector harboring a basta resistance (Hellens et al. 2000; Heidstra et al. 2004). We used three independent T2 lines for GUS analysis and confirmed consistent GUS signals across all three lines.

To compare the expression patterns of *UTP18_pro_* and *mrl-1_pro_*, we constructed two promoter reporter lines: *UTP18_pro_long:3xVENUSnls* and *mrl-1_pro_(2365bp):3xVENUSnls*. Both were gateway cloned into *pGII0229R4R3*. Three independent T3 homozygous lines were used for confocal imaging and by using the same settings we confirmed that the expression patterns were consistent among the three lines.

*mrl-1_pro_(1964bp):GFP-UTP18* and *UTP18_pro_long:GFP-UTP18* complementation vectors were constructed with a FAST-Red selection cassette into *pICSL4723*. For these FAST-Red containing constructs transformed into *mrl-2^+/−^* plants, next generation T1 seeds were selected based on red seed phenotype, sown on soil and subsequently genotyped for *mrl-2^+/−^* heterozygosity by PCR before harvesting the T2 generation. We grew six plants from T2 generation for each (T2-5, T2-16) on soil and genotyped these for *mrl-2^+/−^* heterozygosity and checked the siliques for complementation of the seed abortion phenotype. We also checked the GFP-MRL signals in these ovules by confocal microscopy. Similarly, we checked the siliques in one of the *mrl-1_pro_(1964bp):GFP-UTP18*; *mrl-2*^+/−^ T1 plants (T1-15), and examined the GFP-MRL signals in the (delayed) embryos using the same confocal settings. Three plants from three independent *UTP18 _pro_long:GFP-UTP18; mrl-1* T2 lines were grown in soil of which embryos containing GFP-UTP18 signal were imaged.

*UTP18_pro_short(536bp):UTP18* and *UTP18_pro_long(3218bp):UTP18* were constructed by gateway cloning the respective promoter fragments upstream of the *UTP18* coding sequence in the *pGII0229R4R3* vector. We then transformed the above plasmids into the *mrl-1* mutant and selected positive plants by sowing T1 generation on soil and spraying a 100 mg/L basta solution three times a week. We used T2 seeds for statistics and a homozygous T3 line for imaging.

*DRN_pro_:UTP18* was constructed by Gateway cloning the *DRN* promoter (4,771 bp) upstream of the *MRL* coding sequence in the *pGII0229R4R3* vector. *ATPase_pro_:UTP18* was constructed by Golden Gate cloning, combining FAST-Red selection cassette with the *ATPase_pro_(982bp):UTP18* construct and the endlinker *pICH41744* into the destination binary vector pICSL4723. The ATPase promoter constitutes a 982 bp sequence upstream of the open reading frame of the ATPase gene. The *ATPase_pro_ >> UTP18* was constructed by Golden Gate cloning, combining *Fast-Red*, *UAS_pro_:erGFP*, *ATPase_pro_:GV*, and *UAS_pro_:MRLcds* into *pICSL4723*. We introduced the constructs into the *mrl-1* mutant and determined the penetrance of polyembryony and polycotyledon phenotypes in the T2 lines. One homozygous line (*T3-7-4*) was generated for imaging.

*mrl-1_pro_(2365bp):UTP18* was generated by gateway cloning into *pGII0229R4R3*. Next, we transformed this destination plasmid into *mrl-2*^+/−^, selected T1 plants based on basta resistance. We used two independent T3 homozygous *mrl-1_pro_(2365bp):UTP18*; *mrl-2* lines showing polyembryony for statistics and imaging.

*35S_pro_:GFP-UTP18* was generated by gateway cloning the *UTP18* CDS into pGWB6. Next, we transformed this plasmid into *A. tumefaciens.*

The *UTP18* CRISPR/Cas9 construct *pICSL4723-FASTR-RPS5A_pro_:aCas9-UTP18 sgRNA1,2,3* was generated using Golden Gate cloning (Engler et al. 2014) as described below. CRISPR-P2.0 was used to design spacer sequences 5′-AAAGGGAGAAGCTGCCTGGG, 5′-ATCATGCTAAGCTAAACCCG, and 5′-GATGATGATACTCAGGATGG. These were used to design forward sgRNA primers and amplify corresponding *UTP18sgRNA1*, *UTP18sgRNA2*, and *UTP18sgRNA3* using the *pICH86966-AtU6p:sgRNA_PDS* construct (Addgene plasmid #46966) as a template. PCR products were combined with *AtU6-26* promoter from level 0 plasmid *pICSL90002* (Addgene plasmid #68261) into level 1 vectors *pICH47751*, *pICH47761*, and *pICH47772*, respectively. Subsequently, level 1 vectors harboring sgRNAs were combined with *pICH47732-FAST-Red* and *pICH47742-RPS5A_pro_:aCas9* and the end linker *pICH41800* into level 2 binary vector *pAGM4723* using BpiI restriction-ligation. Plasmids *pICH47732-FAST-Red*, *pICH47742-RPS5A_pro_:aCas9* were generated as described by (Kerstens et al. 2021). A plasmid harboring the Arabidopsis codon optimized aCas9 was kindly provided by the Puchta lab (Fauser et al. 2014). T1 transgenic seeds were selected under a fluorescence binocular (Leica MZ16F).

Inducible CRISPR constructs *pICSL4723-FastRed-G1090_pro_ >> zCas9i-AtU6_26_pro_:UTP18sgRNA1&2* and *pICSL4723-FastRed-PLT7_pro_:zCas9i-AtU6_26_pro_:UTP18sgRNA1&2&3* were generated by Golden Gate cloning and transformed into Col-0. *pICH47732-FASTRed*, *pICH47742-G1090_pro_:XVE, pICH47751-LexA_pro_:zCas9i,* and level 1 vectors harboring sgRNAs (*pICH47761-UTP18sgRNA2* and *pICH47772-UTP18sgRNA1*) were combined with the end-linker *pICH41800* into the level 2 binary vector *pICSL4723* to generate *pICSL4723-FastRed-G1090_pro_ >> zCas9i-AtU6_26_pro_:UTP18sgRNA1&2*. *pICH47732-FASTRed*, *pICH47742-PLT7_pro_:zCas9i*, *pICH47751-UTP18sgRNA1*, *pICH47761-UTP18sgRNA2* and *pICH47772-UTP18sgRNA3* were combined with the end-linker *pICH41800* into the level 2 binary vector pICSL4723 to generate *pICSL4723-FastRed-PLT7_pro_:zCas9i-AtU6_26_pro_:UTP18sgRNA1&2&3.*

### Agrobacterium-mediated infiltration of *Nicotiana benthamiana* leaves

Agrobacterium-mediated infiltration was performed largely according to Diaz-Trivino et al. (2017). In brief: Agrobacterium colonies carrying the *35S_pro_:GFP-UTP18* plasmids were inoculated in 5 mL LB culture (with the appropriated antibiotics) and grown at 28 °C for ∼48 h. The same was done to Agrobacterium carrying the P19 silencing suppressor plasmid. The Agrobacterium cultures were then subcultured (1:100 ratio, v/v) into new 5 mL LB medium with 10 mM 2-(N-morpholine)-ethanesulfonic acid (MES; pH 5.6) and 40 *μ*M acetosyringone. Bacteria were grown at 28 °C until an OD600 of ∼3.0, and then gently pelleted (3,200 × *g*, 10 min). The pellets were resuspended in 10 mM MgCl_2_ to an OD600 = 0.5, and acetosyringone was added to a final concentration of 200 *μ*M. The bacteria were kept at room temperature for at least 3 h without shaking. The Agrobacterium cultures containing the *35S_pro_:GFP-UTP18* and the p19-helper and 10 mM MgCl_2_ were mixed in a v/v proportion of 3:1. With a 5 mL syringe, the mixed solution was infiltrated in young leaves of ∼14-d-old *N. benthamiana* plants. Two days after infiltration, the leaves were observed under the confocal microscope.

### CRISPR/Cas9

CRISPR/Cas9 mediated mutagenesis was used to generate the *mrl-2* knock-out allele in Col-0. Inflorescences of T1 CRISPR/Cas9 mutagenized plants were genotyped for induced mutation by PCR using primers MRL-CRISPRF (5′-GGTTCGGCTTTGTTTCATGT) and MRL-CRISPRR (5′-CACTTCCATCAGAACGCTCA) followed by sequencing. Seeds of mutant plants were selected for absence of the CRISPR/Cas construct followed by next generation genotyping.

### Tissue-specific and inducible CRISPR/Cas9

Inducible CRISPR/Cas9-mediated mutagenesis was used to generate the inducible and tissue-specific knock out of *UTP18* in Col-0 roots. Induction was by transfer to 10 *μ*M β-estradiol (dissolved in DMSO) and DMSO as a control. Five independent T2 lines were checked and T2-1 were used for imaging.

### Histology and microscopy

Imaging of 3 to 7 d seedlings and flowering plants was performed using a Nikon 5300 camera and stereoscopic microscope Nikon SMZ745T. We screened transgenic seeds with the Fast-Red fluorescence marker using the Leica fluorescence binocular (Leica MZ16F). Root systems from the inducible CRISPR experiments were imaged using reflective scanning on an Epson Expression 11,000 XL scanner including an A3 transparency unit. Pollen viability was examined using a simplified Alexander staining (Peterson et al. 2010) on dissected anthers from WT and heterozygous *mrl-2* flowers and imaged using the Zeiss Axioscope.

We visualized GUS activity by staining tissues with a GUS solution containing 10 mM EDTA, 0.1% (w/v) Triton-X100, 1 mM potassium ferrocyanide (K_4_Fe(CN)_6_), 1 mM potassium ferricyanide (K_3_Fe(CN)_6_), 2 mM X-Gluc, and 0.1 M sodium phosphate buffer at pH 7.0. The inflorescences were incubated overnight in GUS solution at 37 °C, seedlings were incubated at 37 °C for 1 h. Images were taken using the Zeiss Axioscope.

To observe embryos within their embryo sac, these were treated with chloral hydrate solution (Lukowitz et al. 1996) and observed using the Zeiss Axioscope with a DIC module. To investigate female gametophyte development we treated ovules with Herr’s solution 2:2:2:2:1 (85% acetic acid:chloral hydrate:clove oil:phenol:xylene) for 4 h (Aulbach-Smith and Herr 1984) and observed treated ovules using the Zeiss Axioscope.

We used the Zeiss LSM 710 and Leica SP8 confocal microscope to detect fluorescence of proteins and cell walls. Cell walls were stained with Renaissance 2,200 (containing 0.1% (v/v) SR2200, 1% (v/v) DMSO, 0.05% (w/v) Triton-X100, 5% (w/v) glycerol, and 4% (w/v) paraformaldehyde in PBS buffer at pH 8.0) (Musielak et al. 2015). SR2200 also successfully stained the nuclei in early embryogenesis.

We excited the green fluorescent protein (GFP) with 488 nm and detected it at a wavelength range of 490 to 595 nm, whereas we visualized Venus by excitation at 514 nm and detection between 515 and 625 nm. SR2200 was excited at 405 nm, and the emission was measured at 410 to 530 nm.

### Protein sequence and phylogenetic analysis

UTP18 protein domains were predicted using the Conserved Domain tool through the NCBI (https://www.ncbi.nlm.nih.gov/Structure/cdd/wrpsb.cgi). Fourteen homologs of the UTP18 protein were obtained using the NCBI Homologene database (https://www.ncbi.nlm.nih.gov/homologene). We aligned the amino acid sequences using MEGA X with the MUSCLE algorithm set (https://www.ebi.ac.uk/Tools/msa/muscle/) to default settings (Kumar et al. 2018), and generated a phylogenetic tree using the neighbor-joining method based on the Jones–Taylor–Thornton model (Jones et al. 1992) (Supplementary Files 3 and 4). To calculate Bootstrap support values, we performed 1,000 replicates of the tree topology. The bootstrap values, representing the percentage of replicate trees where associated taxa clustered together, are shown next to the branches. The branch length representing the evolutionary distances are in the units of the number of amino acid substitutions per site. The rate variation among sites was modeled with a gamma distribution (shape parameter = 1). All ambiguous positions were removed for each sequence pair (pairwise deletion option).

The predicted protein structures of UTP18 homologs were downloaded from AlphaFold (https://alphafold.ebi.ac.uk/).

### *UTP18* expression and DNA gel blot analysis

To determine transcript levels of *UTP18/AT5G14050* and *AT1G01830* throughout development, custom R scripts were used to extract mRNA levels from publicly available datasets and plot them as transcripts per million (TPM) values (https://github.com/michael-nodine/MEERLING). Sample ID, tissue, data accession number, and references are provided in Supplementary Data Set 8.

For RT-qPCR, we extracted total RNA from 7-d-old roots, inflorescences, cauline leaves, stems, and siliques of *mrl-1* and No-0 WT using the Spectrum Plant Total RNA Kit (Sigma) combined with Dnase I treatment. We then reverse-transcribed the purified total RNA using the SuperScript II reverse transcriptase (Thermofisher) and the oligoTrimer. We used the transcribed cDNA as a template for qPCR reactions with Bio-Rad SsoAdvanced Universal SYBR Green Supermix for dTTP assays. The CFX96 real-time PCR system (Bio-Rad) was used to perform qPCR reactions with the UTP18 qPCR-F/R primer pair. *EIF4A1* was used as the reference gene (Supplementary Data Set 9) (Papareddy et al. 2021). The relative expression levels were determined using the 2^−ΔΔ*Ct*^ analysis method. Significance analysis was performed using the two-tailed *t*-test function on the ΔΔ*Ct* values.

To test *UTP18* expression upon inducible CRIPSR, we extracted total RNA from 5 dpi seedlings of two transgenic T2 lines (T2-4, T2-5) harbouring *pICSL4723-FastRed-G1090_pro_ >> zCas9i-AtU6_26_pro_:UTP18sgRNA1&2* and reverse-transcribed it as described above. The cDNA was again used as a template for qPCR using *UTP18* qPCR-F/*UTP18* qPCR-R primer pairs.

To test processing of pre-18S rRNA, we transferred the roots of 5-d-old mock-treated seedlings (T2-4 and T2-5 lines, DMSO plate) to callus-inducing medium (0.5× MS with 1% (w/v) sucrose, 0.5 mg/L 2,4-D and 0.05 mg/L kinetin) for 2 d, followed by transfer to callus-inducing medium containing 10 *μ*M β-estradiol or DMSO. After 9 d, total RNA was extracted from callus, reverse-transcribed, and used it for PCR with the primer pairs P1 and P2. In addition, we used cDNA generated from the induced CRISPR lines as template for PCR with the U1 and U2 primers described in Shi et al. (2005) (Supplementary Data Set 6). An MJ Research PTC-200 Gradient Thermal Cycler was used to carry out PCR reactions. Significant differences in relative expression were determined using the Excel two-tailed *t*-test function (Supplementary Data Set 9). We assumed equal variances between the control (DMSO) group and treatment (β-estradiol) group.

For DNA gel blotting, genomic DNA (20 *µ*g) was digested with HindIII and subsequently separated by 0.7% (w/v) agarose gel electrophoresis followed by Southern transfer to a Supercharge N+ membrane (Schleiger and Schluel). Detection of T-DNA was performed by hybridizing to the ^32^P-labeled Bar gene as a probe (Sambrook et al. 1989).

### Accession numbers

Sequence data from this manuscript are available at the NCBI SRA database (https://www.ncbi.nlm.nih.gov/sra) under the BioProject accession nr PRJNA1009023. Genes mentioned in this manuscript can be found in the GenBank/EMBL data libraries under the following accession numbers: *MRL/UTP18* (At5G14050), *DRN* (AT1G12980), *ATPase* (At5g40010), *IAA12/BDL* (AT1G04550), *TWN2* (AT1G14610), *IYO* (AT4G38440), *TOZ* (AT5G16750), and *PCN* (AT4G07410).

## Supplementary Material

koae087_Supplementary_Data

## Supplementary data

The following materials are available in the online version of this article.

**Supplementary Figure S1**. The *mrl-1* mutant is not associated with the *pCB1* derived T-DNA.

**Supplementary Figure S2**. Phenotypes displayed by the *mrl-1* and the reconstituted *mrl-1* mutant.

**Supplementary Figure S3**. *DR5_pro_-nlsVENUS* reporter expression in WT and *mrl-1* embryo.

**Supplementary Figure S4**. MRL protein sequence and its phylogeny.

**Supplementary Figure S5**. Transcript levels of *UTP18/AT5G14050* and *AT1G01830* throughout development.

**Supplementary Figure S6**. Asynchronous ovule development in heterozygous *mrl-2*^+/−^ plants.

**Supplementary Figure S7**. Embryonic expression mediated by the WT *UTP18* and mutant *mrl-1* promoter.

**Supplementary Figure S8**. Ovule development in WT, *mrl-1* and complemented *mrl-2* mutants.

**Supplementary Figure S9**. Complementation of the *mrl-1* mutant.

**Supplementary Figure S10**. Inducible CRISPR/Cas9 of *UTP18.*

**Supplementary Data Set 1**. Polyembryo and polycotyledon phenotypes.

**Supplementary Data Set 2**. Embryo stages in subsequent siliques in mrl-1 and WT.

**Supplementary Data Set 3**. Suspensor cell numbers related to silique position.

**Supplementary Data Set 4**. Ovule abortion in the *mrl-2* allele siliques.

**Supplementary Data Set 5**. Complementation test.

**Supplementary Data Set 6**. Golden Gate plasmids and primers.

**Supplementary Data Set 7**. Gateway plasmids and primers.

**Supplementary Data Set 8**. Sample ID and references used for Supplementary Fig. S5.

**Supplementary Data Set 9**. RT-qPCR primers and results.

**Supplementary File 1**. *UTP18* (wt) genomic region.

**Supplementary File 2**. *mrl-1* genomic region.

**Supplementary File 3**. UTP18 protein homolog alignment.

**Supplementary File 4**. UTP18 homolog bootstrap tree.

## Data, materials, and software availability

All study data are included in the article and/or Supplementary materials. The Arabidopsis lines and plasmids used in this study will be shared upon request.

## Dive Curated Terms

The following phenotypic, genotypic, and functional terms are of significance to the work described in this paper:

SWA1 Gramene: AT2G47990
SWA1 Araport: AT2G47990

AT1G01830 Gramene: AT1G01830
AT1G01830 Araport: AT1G01830

AT5G14040 Gramene: AT5G14040
AT5G14040 Araport: AT5G14040

EIF4A1 Gramene: At3g13920
EIF4A1 Araport: At3g13920

WOX8 Gramene: AT5G45980
WOX8 Araport: AT5G45980

twn2 Gramene: AT1G14610
twn2 Araport: AT1G14610

SRA Gramene: Sequence Read Archive
SRA Araport: Sequence Read Archive

bHLH49 Gramene: AT1G68920
bHLH49 Araport: AT1G68920

TOZ Gramene: AT5G16750
TOZ Araport: AT5G16750
