# Supplementary material for: Arabidopsis ribosomal RNA processing meerling mutants exhibit suspensor-derived polyembryony due to direct reprogramming of the suspensor
Source: Plant Cell. 2024 Mar 21;36(7):2550–69. doi: 10.1093/plcell/koae087 (PMC11218825; doi:10.1093/plcell/koae087)
Supplement: koae087_Supplementary_Data [file koae087_supplementary_data.zip › Supplemental Figures V11.pdf]

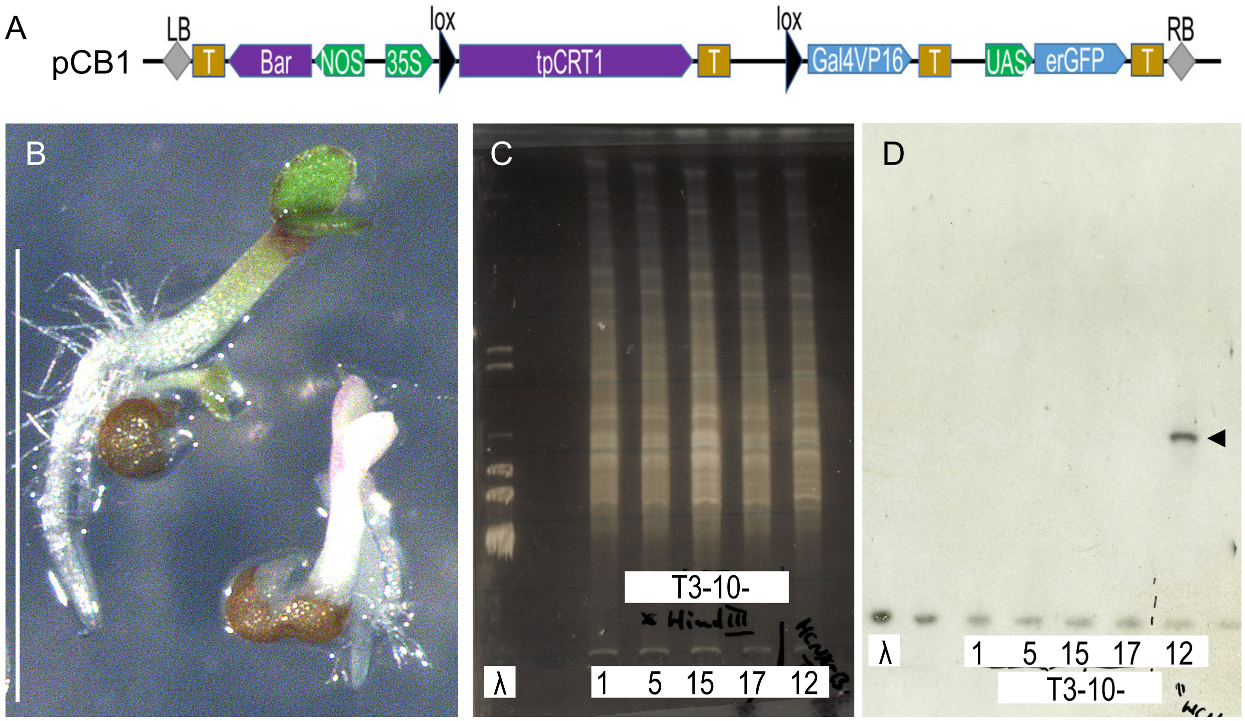

Supplemental Figure S1. The *mrl-1* mutant is not associated with the *pCB1* derived T-DNA (Supports Figure 1).

A. Schematic representation of the *pCB1* T-DNA. Promoters in green, terminators in gold, resistance genes in purple, genes in blue. LB and RB border sequences in grey, loxP recombination sites as triangles.

B. Polyembryonic seeds germinating norflurazon resistant and sensitive seedlings in the progeny of one of the selected lines (T2-10) Scalebar=10mm

C. Digestion pattern of genomic DNA isolated from norflurazon sensitive plants of T3 lines (T3-10-1, 5, 15 and 17) and one norflurazon resistant line (T3-10-12), all showing the polyembryo phenotype. Genomic DNA and a lambda DNA marker was digested with Hind**III**.

D. DNA gel blot of digested DNA in C. Absence of a hybridising band representing the *Bar* gene together with the norflurazon sensitivity indicates that the T-DNA does not correlate with the observed polyembryo phenotype.

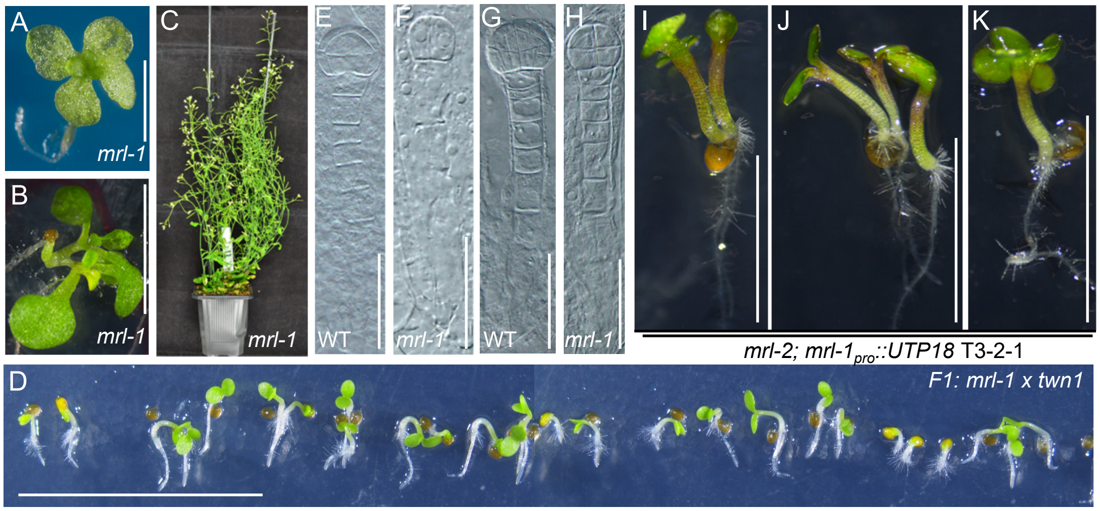

Supplemental Figure S2. Phenotypes displayed by the *mrl-1* and the reconstituted *mrl-1* mutant (Supports Figure 1 and 3).

A. *mrl-1* seedling germinating with 4 cotyledons.

B. *mrl-1* seedling germinating with two shoots connected to a single root.

C. Adult *mrl-1* plant that germinated as a twin seedling.

D. F1 seedlings from a cross between *mrl-1* and *twn1* showing single germinating seedlings only.

E-H. Embryo development is delayed in *mrl-1* mutant. Embryo stages at 48 and 72 hours after hand pollination in WT (E,G) and *mrl-1* (F,H).

I-K. Reconstituted *mrl-1-like* mutant phenotypes in *mrl-2;mrl-1*$_{pro}$*:UTP18* line T3-2-1, germinating seeds forming twins (M), triplets (N), and polycotyledon seedlings (O).

Scale bar: A, 5mm; B, D, I-K: 10mm; E-H: 50 μm.

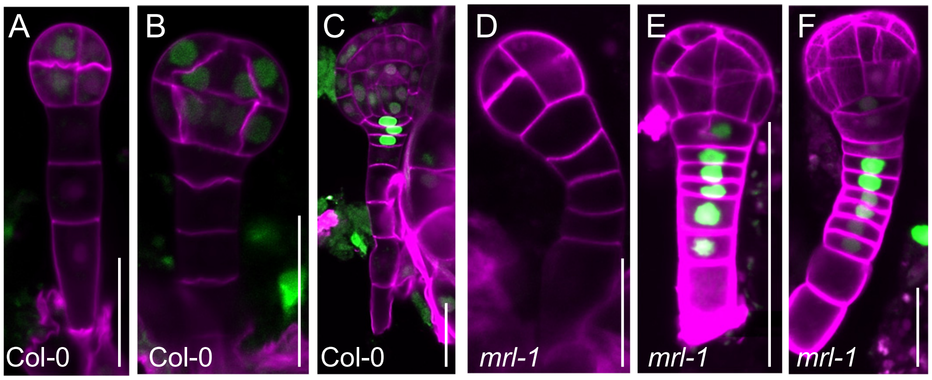

Supplemental Figure S3. *DR5*$_{pro}$*:nlsVENUS* reporter expression in WT and *mrl-1* embryo (Supports Figure 2).

A-C. VENUS signal detected in WT embryo proper of octant (A) and globular (B,C) stage embryos. Highest *DR5* expression in late WT globular stage embryo in the uppermost suspensor cell and the precursors of the quiescent center and columella stem cells (C).

D-F. VENUS signal is absent in early-stage embryo proper in *mrl-1* (D), whereas it ectopically accumulates predominantly in suspensor cells around the globular stage (E,F).

VENUS signal in false green. All confocal imaging was performed using the same settings for WT and *mrl-1*. We checked more than 5 embryos for each stage. Scale bar is 50μm.

A

MSLSQNAPKSKGIKREELKKQYEDV
EDEEEIGSDDDLTRGKRRKTEKEKQ
KLEESELVEMKKLENLIFGSLYSPVT
FGKEEEEEDGSALFHVDRSAVRQIP
DYEDDGDDDEELSDEENGQVVAIRK
GEAAWEDEEEKQINVDIASVNRLRK
LRKEENEGLISGSEYIARLRAHHAKL
NPGTDWARPDSQIVDGESSDDDDT
QDGGVDDILRTNEDLVVKSRGNKLC
AGRLEYSKLVDANAADPSNGPINSV
HFHQNAQLLLTAGLDRRLRFFQIDG
KRNTKIQSIFLEDCPIRKAAFLPNG
SQVIVSGRRKFFYSFDLEKAKFDKIG
PLVGREEKSLEYFEVSQDSNTIAF
VGNEGYILLVSTKTKELIGTLKMNGS
VRSLAFSEDGKHLLSSGGDGQVYV
WDLRTMKCLYKGVDEGSTCGTSLC
SSLNGALFASGTDRGIVNIYKKSEFV
GGKRKPIKTVDNLTSKIDFMKFNHD
AQILAIVSTMNKNSVKLVHVPSLTV
FSNWPPPNSTMHYPRCLDFSPGSG
FMAMGNAAGKVLLYKLHHYQNA

B

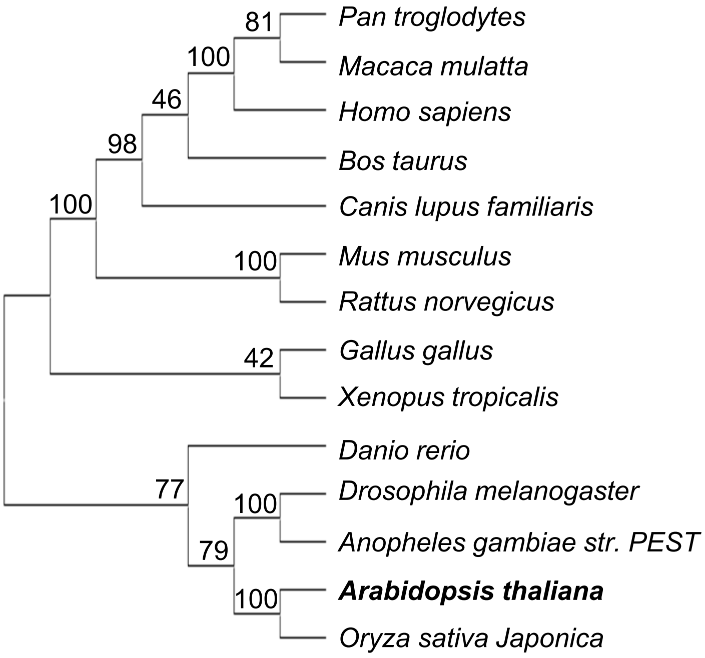

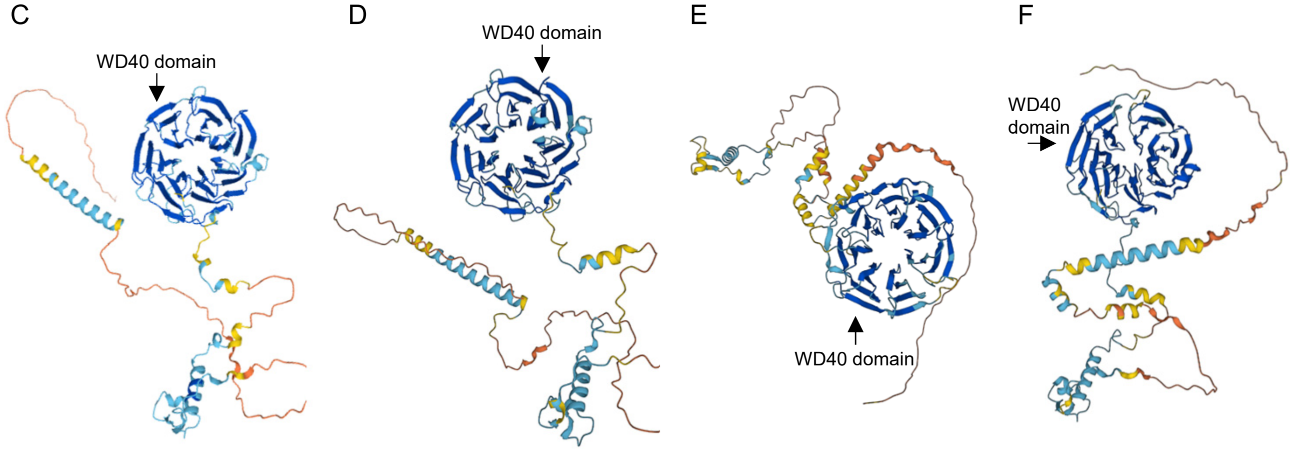

Supplemental Figure S4. UTP18 protein sequence and its phylogeny (Supports Figure 3).

A. Amino acid sequence of the UTP18 protein containing four WD repeats (white letters on black background).

B. Phylogenetic tree showing UTP18 homologs in various species. The branch length represents the evolutionary distances measured in the number of amino acid substitutions per site. Bootstrap support values calculated from 1000 replicates are given at the branch nodes.

C-F. UTP18 protein stucture prediction by AlphaFold for *Arabidopsis thaliana* (C), *Oryza sativa* (D), *Saccharomyces cerevisiae* (E), *Homo sapiens* (F).

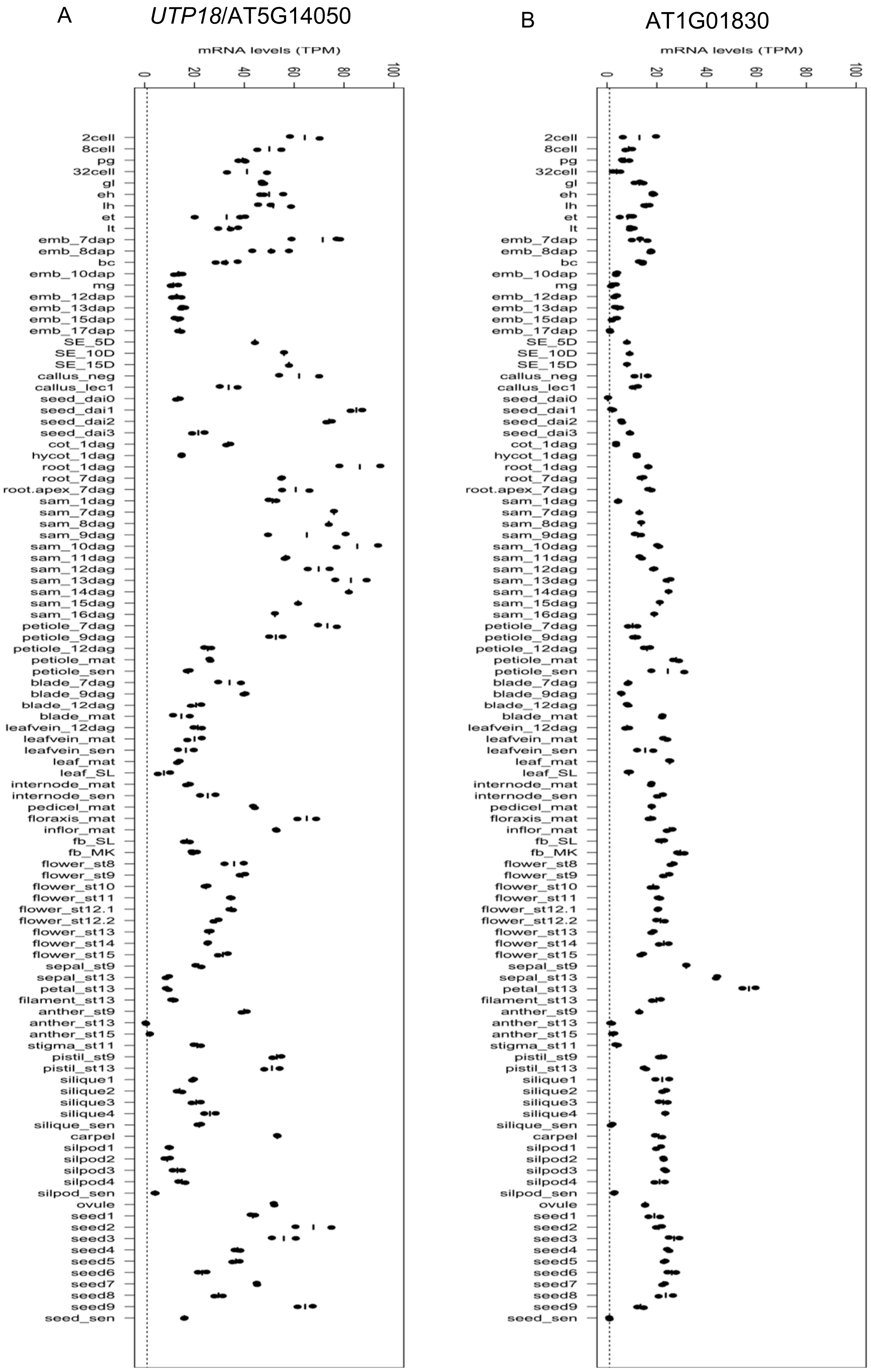

Supplemental Figure S5. Transcript levels of *UTP18/AT5G14050* and *AT1G01830* throughout development (Supports Figure 4). Custom R scripts were used to extract mRNA levels from publicly available datasets and plot them as transcripts per million (TPM) values for *UTP18/AT5G14050* (A) and *AT1G01830* (B). Sample ID, tissue, data accession number and references are provided in Supplemental Data Set 8.

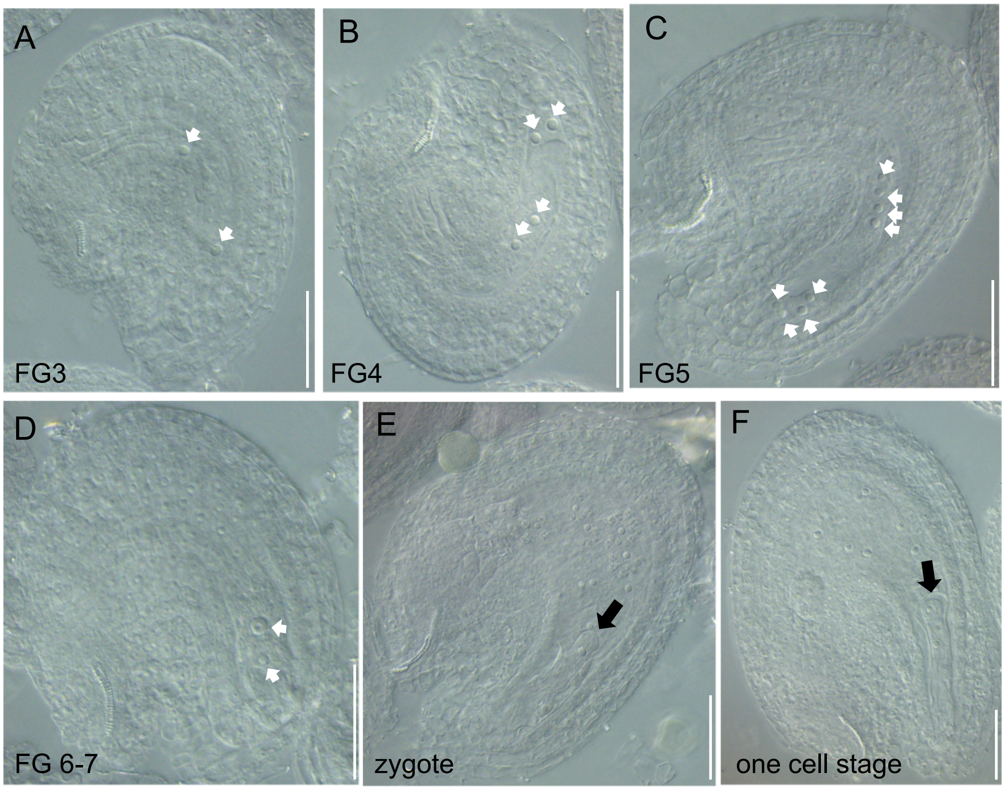

Supplemental Figure S6. Asynchronous ovule development in heterozygous *mrl-2*$^{+/-}$ plants (Supports Figure 4).

A-F. Ovules from the same pistil, stage sl15, showing embryo sacs at different developmental stages from FG3 (A), FG4 (B), FG5 (C), FG6-7 (D) to zygote (E) and one cell stage embryo (F). White arrows point to gametophyte nuclei, black arrow points to embryo. Scale bar is 50µm.

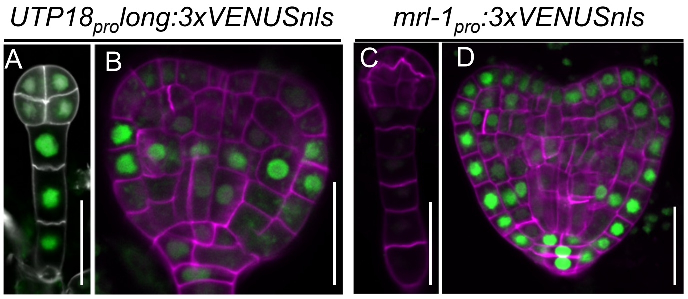

Supplemental Figure S7. Embryonic expression mediated by the WT *UTP18* and mutant *mrl-1* promoter (Supports Figure 3 and 5).

A,B. Transgenic Col-0 embryos at octant (A) and transition stage (B) expressing *UTP18$_{pro}$long:3xVENUSnls.*

C,D. Transgenic Col-0 embryos at dermatogen (C) and early heart stage (D) expressing *mrl-1$_{pro}$:3xVENUSnls.*

Images were generated using the same confocal settings. Images are representative of three homozygous T3 lines of each genotype. At least 5 embryos were examined in independent stages. VENUS in false green. Scale bar is 50 µm.

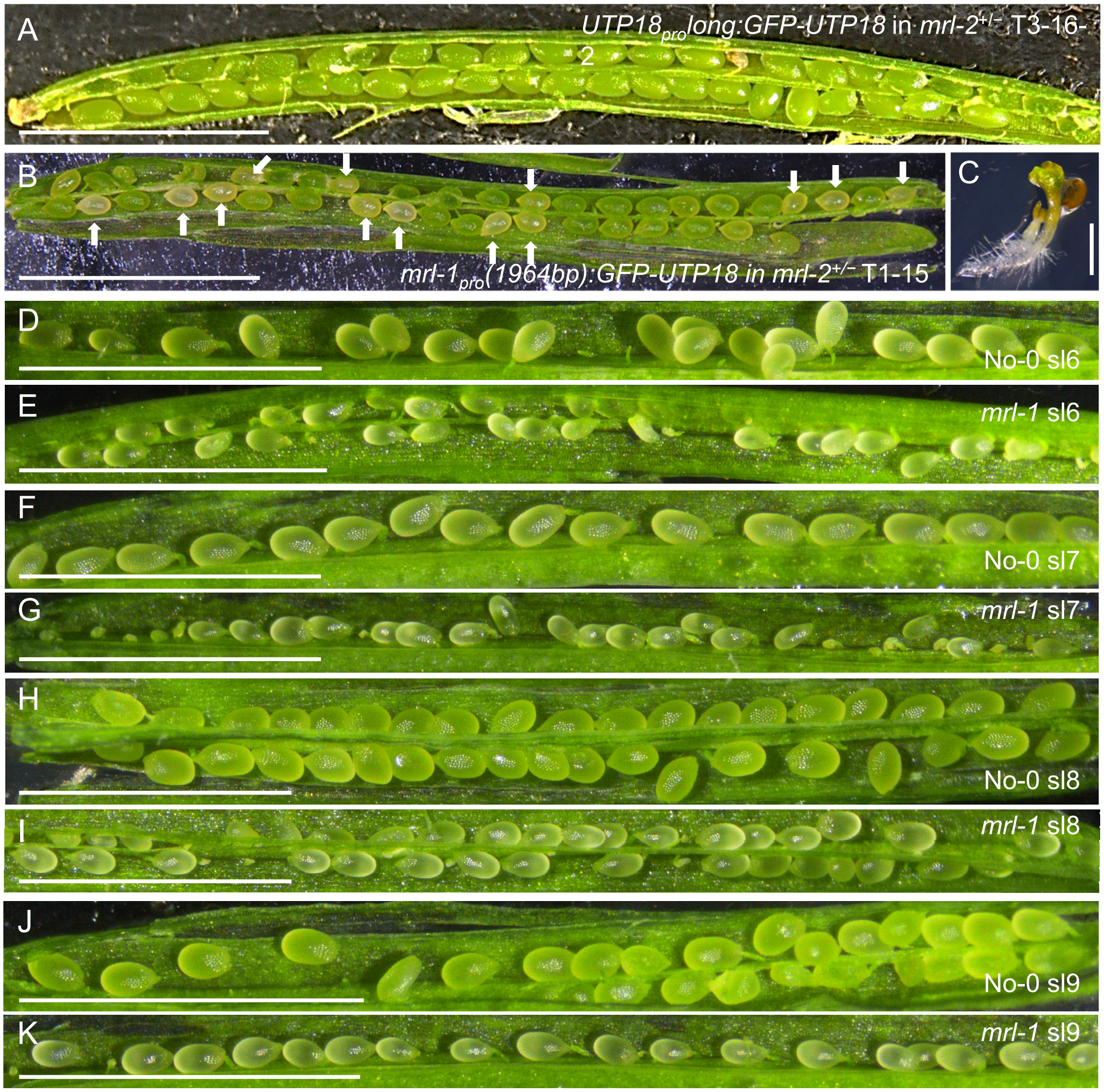

Supplemental Figure S8. Ovule development in WT, *mrl-1* and complemented *mrl-2* mutants (Supports Figure 4 and 5).

A. Dissected silique of *mrl-2*$^{+/-}$;$UTP18_{pro}$*long:GFP-UTP18* transgenic plant (T3-16-2) showing rescue of the seed abortion phenotype. Compare to Fig. 4N.

B. Dissected silique of *mrl-2*$^{+/-}$;*mrl-1*$_{pro}$*(1964bp):GFP-UTP18* transgenic plant (T1-15). The arrows indicate ovules with delayed development.

C. Example of 5 dpg T2-15 twin seedling homozygous for *mrl-1*$_{pro}$*(1964bp):GFP-UTP18* in *mrl-2*$^{+/-}$.

D-K. Comparison of ovule development in No-0 (WT, D,F,H,J) and *mrl-1* (E,G,I,K) siliques, taken from corresponding position of a single inflorescence in each genotype.

Scalebar is 5mm.

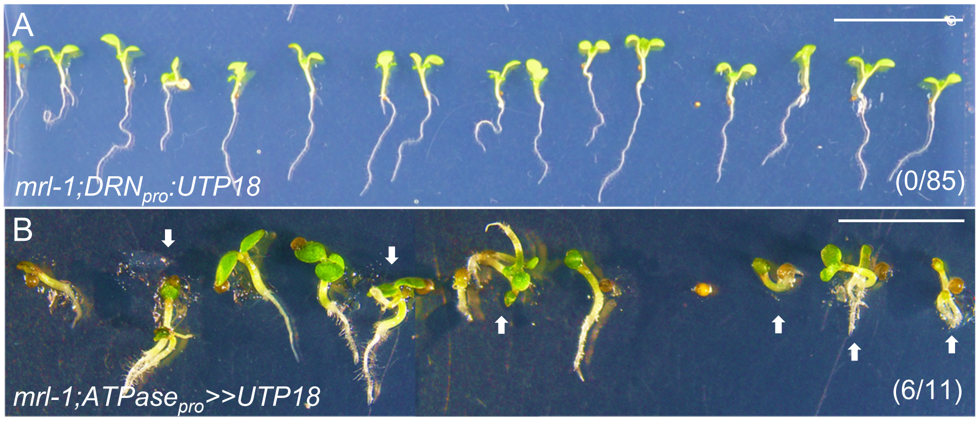

Supplemental Figure S9. Complementation of the *mrl-1* mutant (Supports Figure 5).
A. Five-day old seedlings of a *mrl-1;DRN*$_{pro}$*:UTP18* homozygous line (T3-1-1) showing complementation of the polyembryo phenotype to WT (n=85).
B. Five-day old seedlings of a *mrl-1;ATPase*$_{pro}$*>>UTP18* homozygous line (T3-7-4) indicating maintenance of the *mrl-1* polyembryo phenotype (arrows, n=11).
Scalebar in A is 10mm, in B is 5mm.

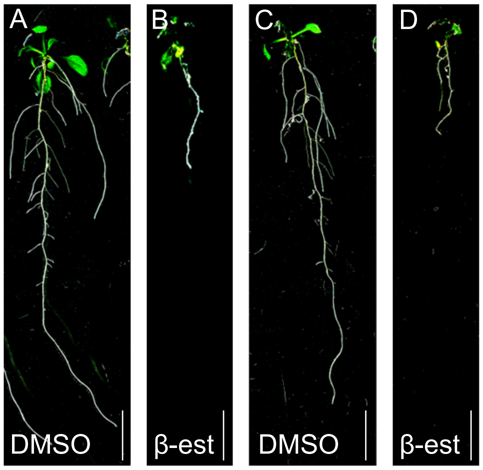

Supplemental Figure S10. Inducible CRISPR/Cas9 of *UTP18* (Supports Figure 6).
A-D. Transgenic 15d old seedlings of lines T2-4 (A, B) and T2-5 (C, D) harboring *G1090*$_{pro}$*>>zCAS9i-AtU6_26*$_{pro}$*:UTP18sgRNA1&2* in Col-0 background. Five-day old seedlings were induced with mock (DMSO, A,C) or 10µM β-estradiol (β-est, B,D) for 9 days.
Scalebar is 10mm.
