# Supplementary material for: Arabidopsis ribosomal RNA processing meerling mutants exhibit suspensor-derived polyembryony due to direct reprogramming of the suspensor
Source: Plant Cell. 2024 Mar 21;36(7):2550–69. doi: 10.1093/plcell/koae087 (PMC11218825; doi:10.1093/plcell/koae087)
Supplement: koae087_Supplementary_Data [file TPC2023RA01122R1_Merged_Decision_Summary_PDF.pdf › TPC2023RA01122R1_Merged_Decision_Summary_PDF.pdf]

THE PLANT CELL

meerling interferes with ribosomal RNA processing and triggers suspensor derived polyembryony in Arabidopsis

Honglei Wang, Luca Santuari, Tristan Wijsman, Guy Wachsman, Hannah Haase, Michael Nodine, Ben Scheres, and Renze Heidstra

Review Timeline:

Submission Date:	20-Nov-2023
Editorial Decision:	15-Jan-2024
Revision Received:	06-Feb-2024
Accepted:	27-Feb-2024

Dr. Renze Heidstra
Wageningen University & Research
Plant Developmental Biology
Wageningen 6708PB
Netherlands

Dear Dr. Heidstra:

We have received reviews of your manuscript entitled "Interfering with ribosomal RNA processing triggers suspensor derived polyembryony in Arabidopsis." Thank you for submitting your best work to The Plant Cell. The editorial board agrees that the work you describe is substantive, falls within the scope of the journal, and may become acceptable for publication, pending revision and potential re-review.

As evident from the reviewer's assessments, two of the consulted experts express an overall positive view of the manuscript, while one exhibits less enthusiasm, which is primarily attributed to the novelty of the described phenotype. Instead of reiterating the reviewer's comments unnecessarily, I would like to draw your attention to the concise feedback provided by reviewer #3. Although the phenotype may not be novel, I, along with two of the reviewers, consider the discovery of the MEERLING mutants associated with this phenotype highly significant. Nevertheless, it is very important to explicitly emphasize the manuscript's novel aspects while addressing the concerns raised by reviewers 1 and 2.

We should stress that we are reluctant to see manuscripts undergoing multiple rounds of revision and would be unlikely to offer you more than one chance to satisfy the reviewers.

Please contact us if there are ambiguous comments or if you wish to discuss the revision.

Given the nature of the comments, we are offering you 60 days from when we have issued this decision to complete the revision. If a revision is not returned within this time frame, and if you have not been granted an extension, we will withdraw the manuscript, which will leave you free to submit the work elsewhere. If you need an extension, we encourage you to contact us at any point before the 60 days have passed.

When you are ready to submit the revised version, please upload a highlighted copy that indicates all changes made in response to the editor and reviewer recommendations. Include an itemized list of all changes made in response to each of the reviewer's suggestions in the "Response to Reviewers" section; please note that reviewers do not have access to your cover letter, nor was this decision letter shared with them.

Thank you for the privilege of reviewing your work. We look forward to receiving your revised manuscript.

Sincerely,

The Plant Cell Editorial Board

Please note the following:

-The Plant Cell now requires authors to complete and submit an author revisions checklist upon submission of a revised manuscript. The aim of the checklist is to aid authors in preparing a high-quality manuscript, facilitate the review and assessment of revised manuscripts, and help to ensure that journal standards are maintained across the board. If your manuscript is accepted, the completed checklist will be published as supplemental material attached to the article online. Please download a copy of the checklist (pdf fillable form) at this link, for submission with your revised manuscript: https://tpc.msubmit.net/html/Author_Revisions_Checklist.pdf.

-Supplemental materials should be restricted to large datasets and tables, presentation of replicates, and validation of reagents, methods, or genotypes. Any data that are used to support the major claims must be in the main manuscript. Supplemental figure legends must indicate what figure in the main manuscript is supported by the supplemental data presented. Please justify how each of the supplemental figures meet the criteria.

-Sampling methods and nature of "biological replicates" should be described precisely (i.e. different plants, parts of plants, pooled tissue, independent pools of tissue, sampled at different times, etc), along with a clear description of

and rationale for any statistical analyses conducted. The reader should know exactly what was sampled; what forms the basis of the calculation of any means and statistical parameters reported. This is also necessary to ensure that proper statistical analysis was conducted.

-Want to add this revision deadline to your calendar? Click below!

-- Reviewer comments:

Reviewer #1 (Comments for the Author):

The manuscript by Wang et al provides genetic evidence supporting the hypothesis that the embryo proper inhibits the developmental potential of suspensor cells. They described the MEERLING gene, which encodes an Arabidopsis UTP18 homolog that localizes in the nucleolus, regulates both male and female gametophyte, embryogenesis and post-embryonic (root) development. Further investigation showed that MEERLING is required for the pre-18S rRNA cleavage process. The manuscript is well written and the figures are clear. This manuscript adds on previous knowledge of the suppressed embryogenic potential ofthe Arabidopsis suspensor provides.

Comments to the authors:

1. The title "Interfering with ribosomal RNA processing triggers suspensor derived polyembryony in Arabidopsis" appears to be an overstatement. Although the authors provide clear evidence that wild-type levels of MEERLING expression are required for timely embryo development and the repression of suspensor-derived polyembryony, there is no link between ribosomal RNA processing and suspensor derived polyembryony. The possibility that MEERLING regulates polyembryony through other unidentified molecular functions can not be excluded.

2. In figure 2R-T, the authors found that expression of the DRN reporter is not only expressed in the embryo proper, but also ectopically expressed in the suspensor cells of /mrl-1/ embryos. However, in figure 5F, the expression of the DRN reporter in /mrl-1/ embryos is wild-type like, without suspensor expression. The authors write in line 391 "First, we confirmed that DRN reporter expression was limited to the embryo proper in mrl1 early embryo development"

Likewise, in figure 2X-Z, the authors show that the expression of the suspensor marker of M0171 was absent from the /mrl-1/ suspensor cells. However, in figure5G, another suspensor marker showed the same expression pattern between /mrl-1/ and WT embryos. Please explain these contradictions. Or do the embryos shown in Fig. 5F-G express a complementation construct?

3. Since /mrl-1/ also shows a seedling phenotype, why didn't the author test pre-18S rRNA cleavage in /mrl-1 /homozygotes seedlings/? /Please explain.

4.line 83ff: "This led to the hypothesis developed in the 1970s... " please add original reference

5. Line 88 "More direct evidence came from laser ablation studies...", please add reference.

6. Line211, "((Radoeva et al., 2016)"

7. I missed a more thorough discussion of what is novel here compared to the previous knowledge that ablation of the embryo proper can induce a second embryo. How do the authors explain that a mere delay in embryo development would do the same? While the mutant embryo stages are trailing the wild-type ones, the same embryo stages in wild type do not cause polyembryony. Is this a question of synchrony between embryo and suspensor development? Or is the polyembrony not caused by the developmental delay of the primary embryo proper at all? It would would be nice to read a bit more about the authors thoughts.

Reviewer #2 (Comments for the Author):

The manuscript "Interfering with ribosomal RNA processing triggers suspensor derived polyembryony in Arabidopsis" characterizes a novel mutation in a gene encoding for a U3 snoRNP component, called MEERLING.
In this specific mutant, genetic rearrangement has led to a novel chimeric gene, changing the expression of At5g14050. Consequently, the gene product accumulates at reduced levels in the embryo proper while still expressed in suspensor cell and at later stages of development.
Reduced level of expression in the embryo proper seem to lead to delayed embryo development and non-cell autonomously trigger secondary embryos in the suspensor.

While this phenomenon is in principle known, this novel mutant shed further light on this enigmatic process.
What strikes me in this context, is the early absence of suspensor-specific marker gene expression in the mutant, indicating that these "suspensor" cells might have never acquired suspensor identity. So, rather than being reprogrammed, one might argue that they have not been programmed in the first place.
These exciting findings raise further questions about suspensor differentiation (and cell fate specification in the early embryo-suspensor structure in general).
The manuscript is written in a clear and easy-to-follow way, and I enjoyed reading it. I especially liked the use of the PLT7 promoter for conditional knock-out of MRL in lateral root primordia. This is a smart way to work with essential genes.
There are a few issues however that need to be addressed:
Major point:
Line 121: I find this very intriguing that expression of suspensor markers that usually turn on around early globular stage (M0171) are entirely absent in mrl-1 mutants. It would be worthwhile to investigate if these basal cells start with the correct identity in the mutant. This seems to be especially important since the authors repeatedly use the term "reprogramming" implying that initially these cells had suspensor identity. Analysis of an early suspensor marker such as WOX8 or WRKY2 would be necessary to answer this question.
Line29: Although stating this correctly later in the text, the authors claim in the abstract that suspensor cells have embryogenic potential until heart stage. This is not correct as embryogenic potential is already lost during mid globular stage and before transition stage.
Minor points:
Line 216: Reprogramming of suspensors around globular stage (as in Rademacher et al., 2012) seem to involve a dedifferentiation step similar to callus induction and might be mechanistically different from ablation experiments at earlier stages, where suspensor cells still have embryogenic potential and might therefore considered to be not yet committed to suspensor identity. A more nuanced discussion of this situation would strengthen the manuscript.
Line 535 In the discussion, the authors state that DR5 activity in suspensor cells in cell ablation experiments would be consistent with formation of twin embryos upon auxin response inhibition.
This is not intuitive to me. The secondary embryo formation upon auxin response inhibition in the suspensor (Rademacher et al., 2012) might be mechanistically different to suspensor-derived embryo formation in cell ablation experiments (and the mrl-1 mutant). It would be good if the authors could elaborate more on this topic at this point of the discussion. Why is inhibition of auxin response in secondary embryo formation consistent with detectable auxin response in mrl-1 suspensors?

Reviewer #3 (Comments for the Author):

This is a clearly written and thoroughly executed manuscript documenting the phenotype of a hypomorphic allele of MRL, a gene required for 18S pre-ribosomal RNA processing. The hypomorphic mrl-1 allele results in formation of twin embryos, with one embryo differentiating from suspensor tissue. The authors uncover the complicated molecular mechanism for reduced MRL expression in the mrl-1 allele, show that null alleles of the MRL gene result in arrest of megagametophyte development, and that loss of MRL in the root causes accumulation of unprocessed 18S pre-rRNA. The authors also show that expression of the wt MRL gene only in the embryo decreases formation of ectopic embryos from mrl-1 suspensors.

With this manuscript, the authors provide additional evidence that an arrest in embryo development leads to formation of ectopic embryos from the suspensor. While everything about this manuscript is well done, the observation that embryo defects can cause ectopic formation of suspensor-derived embryos is not a novel one. The inhibitory effect of the embryo on suspensor-derived embryogenesis is well known and well accepted, both from original papers on this subject (Vernon and Meinke, 1994; Zhang and Somerville, 1997) as well as more recent papers explicitly studying the molecular basis of this phenomenon (Radoeva et al., 2020).

Response to reviewers

We wish to thank the reviewers for their supportive evaluation and constructive critiques. Below we will address all points raised by the editor and for each of the reviewers. We extensively modified the Discussion in line with the reviewers comments and wishes to highlight the novel and significant results obtained in this study. We indicated all relevant changes in the manuscript text in dark red font.

Reviewer #1 (Comments for the Author):

The manuscript by Wang et al provides genetic evidence supporting the hypothesis that the embryo proper inhibits the developmental potential of suspensor cells. They described the MEERLING gene, which encodes an Arabidopsis UTP18 homolog that localizes in the nucleolus, regulates both male and female gametophyte, embryogenesis and post-embryonic (root) development. Further investigation showed that MEERLING is required for the pre-18S rRNA cleavage process. The manuscript is well written and the figures are clear. This manuscript adds on previous knowledge of the suppressed embryogenic potential ofthe Arabidopsis suspensor provides.

Comments to the authors:

1. The title "Interfering with ribosomal RNA processing triggers suspensor derived polyembryony in Arabidopsis" appears to be an overstatement. Although the authors provide clear evidence that wild-type levels of MEERLING expression are required for timely embryo development and the repression of suspensor-derived polyembryony, there is no link between ribosomal RNA processing and suspensor derived polyembryony. The possibility that MEERLING regulates polyembryony through other unidentified molecular functions can not be excluded.

We agree with the reviewer that the title can be overinterpreted. To avoid the direct link between ribosomal RNA processing and polyembryony we changed the title to: "meerling interferes with ribosomal RNA processing and triggers suspensor derived polyembryony in Arabidopsis*"*

2. In figure 2R-T, the authors found that expression of the DRN reporter is not only expressed in the embryo proper, but also ectopically expressed in the suspensor cells of /mrl-1/ embryos. However, in figure 5F, the expression of the DRN reporter in /mrl-1/ embryos is wild-type like, without suspensor expression. The authors write in line 391 "First, we confirmed that DRN reporter expression was limited to the embryo proper in mrl1 early embryo development"

Likewise, in figure 2X-Z, the authors show that the expression of the suspensor marker of M0171 was absent from the /mrl-1/ suspensor cells. However, in figure5G, another suspensor marker showed the same expression pattern between /mrl-1/ and WT embryos. Please explain these contradictions. Or do the embryos shown in Fig. 5F-G express a complementation construct?

*We apologize to for the confusion on the embryo proper and a suspensor reporter expression. Concerning the DRN reporter expression in mrl-1 we find that it is expressed in the embryo proper similar to wild type in early pre-gobular stages. This expression allowed us to use the DRN promoter as driver for the embryo proper expression of MRL in the complementation experiment. We added images of DRN accumulation at an earlier stage in Fig 2 and changed sentences to make the expression patterns more clear as follows at line 203: "*In the F1 WT embryo, *DRN* reporter is specifically expressed in the

embryo proper in agreement with its reported expression pattern (Fig. 2V) (Cole et al., 2009). Prior to the globular stage the *DRN* reporter is only expressed in the embryo proper in *mrl-1*, suggesting that the suspensor identity is initially specified in mrl-1 (Fig. 2W). However, in addition to embryo proper expression, we observed ectopic expression in the transverse dividing suspensor cells around the globular stage (Fig. 2X,Y)"

Indeed, DRN expression from globular stage onward is also observed in the mrl-1 suspensor as visualized in Fig 2X,Y. To make this more clear we changed the sentence at line 401 to: "DRN reporter expression was limited to the embryo proper in *mrl-1* early embryo development before its ectopic expression from globular stage onward (Fig. 2W-Y), allowing its use in complementation experiments." *In addition we adapted Figure 5 to remove the DNR expression panel since the driver marker expression is now provided earlier in Fig 2*

Concerning the suspensor markers, the M0171 marker expression is not initiated in mrl-1 mutant suspensor but it is important to note that the ATPase marker is. Normally the M0171 marker becomes expressed from octant stage onward. Therefore, we tested the ATPase suspensor marker that is expressed from at least four-cell stage onward (Radoeva et al., 2020) and found that it is also expressed in the mrl-1 mutant. Together this indicates that suspensor fate is initially present but becomes partially compromised from around octant stage on. The fact that the ATPase marker is present allows its use for the complementation experiment summarized in Fig 5F. To clarify this point we added images of $ATPase_{pro}$*>>erGFP marker expression in wild-type and mrl-1 mutant in Fig 2(R-U). We added/changed the sentences describing these results at line 216:* "In addition, we tested the $ATPase_{pro}$ suspensor marker that was reported to be expressed from at least the four cell stage onwards (Radoeva et al., 2020). We confirmed its expression in the wild-type and *mrl-1* suspensor (Fig 2R-U). The correct setup of the *DRN* marker expression together with the $ATPase_{pro}$ expression pattern indicate that suspensor fate is initially specified and becomes (partially) lost during early embryogenesis after which ectopic expression of embryo proper fate is followed by suspensor division"

We adapted the sentence mentioning the ATPase driver in line 409 as follows: "To drive MRL expression in the suspensor we used the *ATPase* promoter (Radoeva et al., 2020), that is active in the *mrl-1* suspensor (Fig. 2T,U)."

3. Since /mrl-1/ also shows a seedling phenotype, why didn't the author test pre-18S rRNA cleavage in /mrl-1 /homozygotes seedlings/? /Please explain.

The reason for not testing the pre-18S rRNA cleavage in hypomorphic mrl-1 seedlings is because of their normal development into fully adult plants. This in contrast to the severe developmental defects and inability to grow in the induced knockout (tissues). In addition, this experiment allowed us to mimic the conditions and setup used by Shi et al (2005) that used RNAi to lower overall levels of a UTP15 homolog. We added a sentence to clarify this point at line 457: "To investigate a similar function for MRL as a UTP18 homolog, we used the conditional mutant strategy to induce loss of gene function and subsequently test pre-rRNA cleavage in the affected tissues."

4.line 83ff: "This led to the hypothesis developed in the 1970s… " please add original reference

We added the original reference by Sussex et al (1973).

5. Line 88 "More direct evidence came from laser ablation studies…", please add reference.

Thank you for pointing this out. We added references Gooh et al. (2015) and Liu et al. (2015), whose ablation studies showed that damage to the apical cell that is formed after the asymmetric division of zygote can induce the basal cell reprogramming.

6. Line211, "((Radoeva et al., 2016)"

We removed the double bracket

7. I missed a more thorough discussion of what is novel here compared to the previous knowledge that ablation of the embryo proper can induce a second embryo. How do the

authors explain that a mere delay in embryo development would do the same? While the mutant embryo stages are trailing the wild-type ones, the same embryo stages in wild type do not cause polyembryony. Is this a question of synchrony between embryo and suspensor development? Or is the polyembrony not caused by the developmental delay of the primary embryo proper at all? It would would be nice to read a bit more about the authors thoughts.

In agreement with the reviewer (and reviewer #2) we provide more discussion on the polyembryony in mrl-1 and how this elaborates on our current knowledge. In short, we indicate that interfering with components involved in rRNA processing may not only cause delayed development but can also have an effect on the translatome composition as observed from mammalian studies and we added a sentence to this effect in the Discussion (line 511). We discuss that the high penetrance of the mrl-1 phenotype allows us to pinpoint the timing of reprogramming and secondary embryo formation at around octant stage (line 574 and following). And we discuss on the polyembryony observed in mrl-1 and twn1 as a distinct clade of mutants where suspensor derived embryo formation is not depending on embryo proper damage, and picture a scenario involving active signalling between embryo proper and suspensor as being essential for progression of normal embryo development (line 583 and following). As a result the suspensor reprograms and ectopic embryo fate is established in a process different from the reprogramming observed upon interfering with auxin response or ectopic transcription factor expression. We bring the case of maternal versus embryonic signalling via WIP proteins acting through hub proteins to control root development under favourable or adverse conditions as an example of synchronising development between tissues. We propose how mrl-1 can be used to investigate such a signalling process in future experiments. We provide more discussion in response to reviewer #2 (see below).

Reviewer #2 (Comments for the Author):

The manuscript "Interfering with ribosomal RNA processing triggers suspensor derived polyembryony in Arabidopsis" characterizes a novel mutation in a gene encoding for a U3 snoRNP component, called MEERLING.
In this specific mutant, genetic rearrangement has led to a novel chimeric gene, changing the expression of At5g14050. Consequently, the gene product accumulates at reduced levels in the embryo proper while still expressed in suspensor cell and at later stages of development.
Reduced level of expression in the embryo proper seem to lead to delayed embryo development and non-cell autonomously trigger secondary embryos in the suspensor.
While this phenomenon is in principle known, this novel mutant shed further light on this enigmatic process.
What strikes me in this context, is the early absence of suspensor-specific marker gene expression in the mutant, indicating that these "suspensor" cells might have never acquired suspensor identity. So, rather than being reprogrammed, one might argue that they have not been programmed in the first place.
These exciting findings raise further questions about suspensor differentiation (and cell fate specification in the early embryo-suspensor structure in general).
The manuscript is written in a clear and easy-to-follow way, and I enjoyed reading it. I especially liked the use of the PLT7 promoter for conditional knock-out of MRL in lateral root primordia. This is a smart way to work with essential genes.
There are a few issues however that need to be addressed:

Major point:
Line 121: I find this very intriguing that expression of suspensor markers that usually turn on around early globular stage (M0171) are entirely absent in mrl-1 mutants. It would be worthwhile to investigate if these basal cells start with the correct identity in the mutant. This seems to be especially important since the authors repeatedly use the term "reprogramming"

implying that initially these cells had suspensor identity. Analysis of an early suspensor marker such as WOX8 or WRKY2 would be necessary to answer this question.

Indeed, we were similarly intrigued by the failure of M0171 expression initiation in the suspensor of mrl-1 mutants. As reported, the M0171 becomes expressed from octant stage on. Therefore we tested the ATPase reporter whose expression was reported to be strong in suspensor cells from at least the four-cell stage onwards (Radoeva et al., 2020). This reporter also marked the suspensor of the twn1 mutant until divisions leading to embryonic structures (Radoeva et al., 2020). Therefore, we used this reporter as a proxy for suspensor identity. This reporter reliably marked the suspensor of the mrl-1 mutant and we have now included more images of its expression in WT and mrl-1 in Fig 2(R-U). In addition, we find that the DRN marker is initially correctly setup in the embryo proper in mrl-1 and does not localize to the mutant suspensor cells until globular stage. Together with the initial normal morphology and the ATPase marker expression, this indicates that the suspensor fate is partially compromised from octant stage onwards. This also explains our use of the word "reprogramming" when talking about suspensor derived embryos. We added/changed sentences to this effect as follows at line 205: "Prior to the globular stage the DRN reporter is only expressed in the embryo proper in *mrl-1*, suggesting that the suspensor identity is initially specified in mrl-1 (Fig. 2W). However, in addition to embryo proper expression, we observed ectopic expression in the transverse dividing suspensor cells around the globular stage (Fig. 2X,Y)." And line 216: "In addition, we tested the *ATPase*$_{pro}$ suspensor marker that was reported to be expressed from at least the four cell stage onwards (Radoeva et al., 2020). We confirmed its expression in the wild-type and *mrl-1* suspensor (Fig 2R-U). The correct setup of the *DRN* marker expression together with the *ATPase*$_{pro}$ expression pattern indicate that suspensor fate is initially specified and becomes (partially) lost during early embryogenesis after which ectopic expression of embryo proper fate is followed by suspensor division"

In addition we provide our reasoning above on the reprogramming aspect in the Discussion in lines 583 and following.

Line29: Although stating this correctly later in the text, the authors claim in the abstract that suspensor cells have embryogenic potential until heart stage. This is not correct as embryogenic potential is already lost during mid globular stage and before transition stage.

We modified this phrase in the abstract to: "Until globular stage the suspensor maintains embryonic potential and can form embryos in absence of the developing embryo proper."

Minor points:

Line 216: Reprogramming of suspensors around globular stage (as in Rademacher et al., 2012) seem to involve a dedifferentiation step similar to callus induction and might be mechanistically different from ablation experiments at earlier stages, where suspensor cells still have embryogenic potential and might therefore considered to be not yet committed to suspensor identity. A more nuanced discussion of this situation would strengthen the manuscript.

We agree with the reviewer that we could have been more outspoken in our descriptions and discussion. We adjusted the sentence (now line 223) to indicate the initially similar phenotypic response observed in mrl-1 and ectopic auxin response inhibition, as a reason to investigate the DR5 auxin response marker. We agree with the reviewer that the reprogramming appears different to that observed upon ectopic auxin response inhibition and add a statement to that effect in the last sentence of this paragraph (line 237). In addition, we elaborate on these and our findings in the discussion, lines 583 and following. In short, we propose the observed phenotypic events in ablation studies and the mrl-1 mutant represent a similar mechanism of reprogramming whereby suspensor cells first change fate followed by division towards embryo formation. As opposed to the auxin response inhibition and ectopic transcription factor studies, and we agree with the reviewer that in these cases the reprogramming resembles callus derived somatic embryogenesis whereby suspensor cells first start to divide followed by adopting embryonic markers and outgrowth into somatic embryos.

Line 535 In the discussion, the authors state that DR5 activity in suspensor cells in cell ablation experiments would be consistent with formation of twin embryos upon auxin response inhibition.
This is not intuitive to me. The secondary embryo formation upon auxin response inhibition in the suspensor (Rademacher et al., 2012) might be mechanistically different to suspensor-derived embryo formation in cell ablation experiments (and the mrl-1 mutant). It would be good if the authors could elaborate more on this topic at this point of the discussion. Why is inhibition of auxin response in secondary embryo formation consistent with detectable auxin response in mrl-1 suspensors?

The reviewer is correct. The sentence stating the Rademacher paper was misplaced. Indeed, the mechanism is not similar and we provide a more elaborate discussion to this effect in lines 583 and following, as summarized above with the previous point raised by this reviewer. There we propose that polyembryony in mrl-1 mutants resembles the reprogramming observed in the ablation studies, setting it apart from the reprogramming mechanism observed upon ectopic transcription factor or auxin response inhibition studies.

Reviewer #3 (Comments for the Author):

This is a clearly written and thoroughly executed manuscript documenting the phenotype of a hypomorphic allele of MRL, a gene required for 18S pre-ribosomal RNA processing. The hypomorphic mrl-1 allele results in formation of twin embryos, with one embryo differentiating from suspensor tissue. The authors uncover the complicated molecular mechanism for reduced MRL expression in the mrl-1 allele, show that null alleles of the MRL gene result in arrest of megagametophyte development, and that loss of MRL in the root causes accumulation of unprocessed 18S pre-rRNA. The authors also show that expression of the wt MRL gene only in the embryo decreases formation of ectopic embryos from mrl-1 suspensors.

With this manuscript, the authors provide additional evidence that an arrest in embryo development leads to formation of ectopic embryos from the suspensor. While everything about this manuscript is well done, the observation that embryo defects can cause ectopic formation of suspensor-derived embryos is not a novel one. The inhibitory effect of the embryo on suspensor-derived embryogenesis is well known and well accepted, both from original papers on this subject (Vernon and Meinke, 1994; Zhang and Somerville, 1997) as well as more recent papers explicitly studying the molecular basis of this phenomenon (Radoeva et al., 2020).

We appreciate the points raised by the reviewer, but also ask the reviewer to view our replies to the other reviewers comments, and we recognize that we should have been more outspoken in our discussion of the results. Importantly, we now elaborate in the discussion on the evidence from our studies and propose a different mechanism of reprogramming as compared to the studies of Radoeva et al (2019 and 2020). The order of events leading to suspensor derived embryogenesis are in agreement with those observed in the ablation studies, first a fate change in suspensor identity followed by divisions leading to embryo formation as opposed to a sequence of events resembling somatic embryogenesis. The penetrance and phenotype of the mrl-1 mutant further provides important evidence for active signalling between embryo proper and suspensor and the timing of reprogramming. As such we discuss how it also provides a tool to investigate this signalling.

Dear Dr. Renze Heidstra:

We are pleased to inform you that your paper entitled "meerling interferes with ribosomal RNA processing and triggers suspensor derived polyembryony in Arabidopsis" has been accepted for publication in The Plant Cell, pending a final editorial review by a science editor. At this stage, your manuscript will be evaluated by a science editor with respect to its presentation of scientific content, compliance with journal policies, and presentation for a broad readership. The Plant Cell has contracted with Plant Editors (planteditors.com) to provide this service to our authors, and you will soon receive additional information on this process.

Please note that each author needs to link their ORCID identifier to their account in the system before your manuscript can be published. If any authors do not have an ORCID linked to their account, they will receive a message with a link to complete this task. Please ensure that ALL of your coauthors have completed this task as soon as possible.

ASPB offers an OPEN option that allows authors to have their online articles available for free to all users immediately upon publication. For more information about the ASPB OPEN option, refer to the Final Submission Checklist Form.

The Plant Cell and The Arabidopsis Information Resource (TAIR) are collaborating to collect functional annotation data about Arabidopsis genes from authors. This includes information about the gene's molecular function (e.g., kinase activity, ATP synthetase activity), the biological process/es it is involved in (e.g., endosperm development, threonine biosynthesis), its subcellular location (e.g., nucleus, ER), anatomical or developmental expression pattern (e.g., leaf, ovule, flower stage 10, seedling stage), or its partner in a protein-protein interaction (e.g., AT1G01010 interacts with AT1G01020).

If your paper contains results falling into one or more of these categories for Arabidopsis genes, we request that you now submit these data for inclusion in TAIR by filling in the form provided at the following URL: https://www.arabidopsis.org/doc/submit/functional_annotation/123. If you need further clarification on what types of data can be submitted please contact curator@arabidopsis.org.

Finally, we encourage your submission of artwork for the journal cover. Monthly issues will have an online cover image and selected covers will be used for posters, other promotional items, and "wallpaper" for mobile devices. For more information, refer to Cover Submission in the Instructions for Authors https://tpc.msubmit.net/cgi-bin/main.plex?form_type=display_auth_instructions.

We look forward to seeing your paper published.

Sincerely,

The Plant Cell Editorial Board

=====
IMPORTANT REMINDER: PEER REVIEW REPORTS
=====

If you opted to publish a peer review report along with your article during the original submission process, it will be prepared by the editorial staff and publicly posted with your manuscript, inside the zip file that contains any other supplemental material. As a reminder, the peer review report is a public record of all comments from editors and reviewers, as well as your prior responses, as you received them in the decision letters for each draft of your manuscript. If you agreed to publish this report and have changed your mind, or are not sure if you selected this option, please contact the editorial office as soon as possible before signing the license agreement from our publisher.

=====
REVIEWER COMMENTS
=====

Reviewer #2 (Comments for the Author):

The authors have carefully considered and addressed my concerns in their revised manuscript. I see no further issues that need

to be addressed.

----FOR ASPB OFFICE USE ONLY (DO NOT EDIT)----
MSID: 37138
Corresponding Author: Dr. Heidstra
